# Spinal Sensorimotor Circuits Play a Prominent Role in Hindlimb Locomotor Recovery after Staggered Thoracic Lateral Hemisections but Cannot Restore Posture and Interlimb Coordination during Quadrupedal Locomotion in Adult Cats

**DOI:** 10.1523/ENEURO.0191-23.2023

**Published:** 2023-06-21

**Authors:** Johannie Audet, Sirine Yassine, Charly G. Lecomte, Stephen Mari, Félix Soucy, Caroline Morency, Angèle N. Merlet, Jonathan Harnie, Claudie Beaulieu, Louis Gendron, Ilya A. Rybak, Boris I. Prilutsky, Alain Frigon

**Affiliations:** 1 Department of Pharmacology-Physiology, Faculty of Medicine and Health Sciences, Centre de Recherche du Centre Hospitalier Universitaire de Sherbrooke, Université de Sherbrooke, Sherbrooke, Quebec J1H 5N4, Canada; 2 Department of Neurobiology and Anatomy, College of Medicine, Drexel University, Philadelphia, PA 19129; 3 School of Biological Sciences, Georgia Institute of Technology, Atlanta, GA 30332

**Keywords:** cat, central pattern generator, interlimb coordination, locomotion, spinal cord injury

## Abstract

Spinal sensorimotor circuits interact with supraspinal and peripheral inputs to generate quadrupedal locomotion. Ascending and descending spinal pathways ensure coordination between the forelimbs and hindlimbs. Spinal cord injury (SCI) disrupts these pathways. To investigate the control of interlimb coordination and hindlimb locomotor recovery, we performed two lateral thoracic hemisections on opposite sides of the cord (right T5–T6 and left T10–T11) at an interval of approximately two months in eight adult cats. In three cats, the spinal cord was transected at T12–T13. We collected electromyography (EMG) and kinematic data during quadrupedal and hindlimb-only locomotion before and after spinal lesions. We show that (1) cats spontaneously recover quadrupedal locomotion following staggered hemisections but require balance assistance after the second one, (2) coordination between the forelimbs and hindlimbs displays 2:1 patterns (two cycles of one forelimb within one hindlimb cycle) and becomes weaker and more variable after both hemisections, (3) left-right asymmetries in hindlimb stance and swing durations appear after the first hemisection and reverse after the second, and (4) support periods reorganize after staggered hemisections to favor support involving both forelimbs and diagonal limbs. Cats expressed hindlimb locomotion the day following spinal transection, indicating that lumbar sensorimotor circuits play a prominent role in hindlimb locomotor recovery after staggered hemisections. These results reflect a series of changes in spinal sensorimotor circuits that allow cats to maintain and recover some level of quadrupedal locomotor functionality with diminished motor commands from the brain and cervical cord, although the control of posture and interlimb coordination remains impaired.

## Significance Statement

Coordinating the limbs during locomotion depends on pathways in the spinal cord. We used a spinal cord injury (SCI) model that disrupts communication between the brain and spinal cord by sectioning half of the spinal cord on one side and then about two months later, half the spinal cord on the other side at different levels of the thoracic cord in cats. We show that despite a strong contribution from neural circuits located below the second spinal cord injury in the recovery of hindlimb locomotion, the coordination between the forelimbs and hindlimbs weakens and postural control is impaired. We can use our model to test approaches to restore the control of interlimb coordination and posture during locomotion after spinal cord injury.

## Introduction

Terrestrial locomotion in mammals involves complex dynamic interactions between spinal circuits, supraspinal signals and peripheral sensory inputs [for review, see Rossignol et al., 2006; Frigon, 2017; Frigon et al., 2021). Musculoskeletal properties also contribute in stabilizing quadrupedal locomotion and can offset some loss in neural communication between the brain/cervical cord and the lumbar cord after spinal cord injury (SCI; Audet et al., 2022). After complete spinal thoracic transection, hindlimb locomotion recovers in various mammals, including mice, rats, cats, and dogs (Shurrager and Dykman, 1951; Barbeau and Rossignol, 1987; Bélanger et al., 1996; De Leon et al., 1998, 1999; Leblond et al., 2003; Cha et al., 2007; Alluin et al., 2011; Sławińska et al., 2012; Harnie et al., 2019). This recovery involves the locomotor central pattern generator (CPG) that interacts with sensory feedback from the hindlimbs (Brown, 1911; Grillner and Shik, 1973; Grillner and Zangger, 1979; Forssberg et al., 1980; Barbeau and Rossignol, 1987; McCrea and Rybak, 2008; Rossignol and Frigon, 2011; Kiehn, 2016; Grillner and El Manira, 2020; Frigon et al., 2021). Decerebrate cats with a high cervical (C1–C2) transection (i.e., high spinal cats) express quadrupedal locomotion on a treadmill with pharmacology (Miller and van der Meché, 1976; Miller et al., 1977). However, these studies did not measure the phasing between the forelimbs and hindlimbs. As such, we do not know whether forelimb and hindlimb movements were coordinated. The isolated spinal cord of neonatal rats also produces quadrupedal-like rhythmic activity, as demonstrated by recording the activity from cervical and lumbar roots during drug-induced fictive locomotion (Juvin et al., 2005, 2012). In these neonatal rat preparations, the activity recorded from cervical and lumbar roots is coordinated, consistent with propriospinal pathways coupling cervical and lumbar CPGs (Juvin et al., 2005). Similar demonstrations have not been made in adult preparations and as stated, in high spinal decerebrate cats during treadmill locomotion. In real locomotion on a treadmill or overground, the loss of supraspinal inputs and postural control might prevent coordinated forelimb and hindlimb movements.

Lumbar sensorimotor circuits also play a prominent role in hindlimb locomotor recovery following incomplete SCI (Barrière et al., 2008, 2010). Barrière et al. (2008) performed a dual-lesion paradigm, consisting of a lateral hemisection at T10–T11 followed by complete spinal transection at T12–T13. Instead of taking the minimum two to three weeks of treadmill locomotor training usually required, hindlimb locomotion was expressed the day after transection. Thus, after incomplete SCI, plasticity within lumbosacral circuits allowed them to function without motor commands originating from above the transection. The lumbar locomotor CPG likely contributes to hindlimb locomotor recovery after other types of incomplete SCIs.

Another dual spinal lesion paradigm involves performing two lateral hemisections on opposite sides of the cord at different levels (i.e., staggered hemisections) to determine whether neural communication remains possible between cervical and lumbosacral levels (Ingebritsen, 1933; Kato et al., 1984, 1985; Stelzner and Cullen, 1991; Courtine et al., 2008; van den Brand et al., 2012; Cowley et al., 2015). Kato et al. (1984) performed two types of staggered hemisections in adult cats, low thoracic followed by mid-thoracic and high cervical followed by mid-thoracic. In the two types of staggered hemisection paradigms, new fore-hind coordination patterns emerged, with one forelimb performing two cycles within one hindlimb cycle, or a 2:1 fore-hind coordination, with no apparent consistent phasing between the forelimbs and hindlimbs during overground locomotion. These results indicate that the spinal locomotor CPGs controlling the forelimbs, located at low cervical/upper thoracic segments (Ballion et al., 2001; Yamaguchi, 2004), operated at a different rhythm and independently from those controlling the hindlimbs, located at upper to mid-lumbar spinal segments (Cazalets et al., 1995; Kiehn and Kjaerulff, 1998; Marcoux and Rossignol, 2000; Kiehn and Butt, 2003; Langlet et al., 2005). However, Kato et al. (1984) did not separate cycles with 1:1 (equal number of cycles in the forelimbs and hindlimbs) and 2:1 (two forelimb cycles within a hindlimb cycle) fore-hind coordination. Studies in intact and single-hemisected cats have shown that step-by-step phasing between the forelimbs and hindlimbs can remain consistent despite 2:1 coordination during treadmill locomotion (Thibaudier et al., 2013, 2017; Thibaudier and Frigon, 2014).

The purpose of the present study was to determine how staggered hemisections affected the control of interlimb coordination and the recovery of hindlimb locomotion. We hypothesize that fore-hind coordination is lost following the second hemisection because of the disruption of direct communication between cervical and lumbar levels. We also hypothesize that spinal sensorimotor circuits play a prominent role in the recovery of hindlimb locomotion following staggered hemisections.

## Materials and Methods

### Ethical approval

The Animal Care Committee of the Université de Sherbrooke approved all procedures in accordance with policies and directives of the Canadian Council on Animal Care (Protocol 442–18). Current data were obtained from eight adult cats (more than one year of age at the time of experimentation), four females and four males, weighing between 4.1 and 6.5 kg (5.3 ± 1.0). Before and after the experiments, cats were housed and fed in a dedicated room within the animal care facility of the Faculty of Medicine and Health Sciences at the Université de Sherbrooke. Our study followed ARRIVE guidelines for animals studies (Percie du Sert et al., 2020). As part of our effort to reduce the number of animals used in research, all cats participated in other studies to answer different scientific questions, some of which have been published (Lecomte et al., 2022, 2023; Merlet et al., 2022).

### General surgical procedures

Surgical procedures were performed under aseptic conditions with sterilized equipment in an operating room, as described previously (Hurteau et al., 2017; Harnie et al., 2019, 2021; Audet et al., 2022). Before surgery, cats were sedated with an intramuscular injection of butorphanol (0.4 mg/kg), acepromazine (0.1 mg/kg), and glycopyrrolate (0.01 mg/kg). Ketamine/diazepam (0.05 ml/kg) was then injected intramuscularly for induction. Cats were anesthetized with isoflurane (1.5–3%) delivered in O_2_, first with a mask and then with an endotracheal tube. During surgery, we adjusted isoflurane concentration by monitoring cardiac and respiratory rates, by applying pressure to the paw (to detect limb withdrawal), by assessing the size and reactivity of pupils and by evaluating jaw tone. We shaved the animal’s fur (back, stomach, forelimbs and hindlimbs) using electric clippers and cleaned the skin with chlorhexidine soap. Cats received a continuous infusion of lactated Ringers solution (3 ml/kg/h) during surgery through a catheter placed in a cephalic vein. A rectal thermometer monitored body temperature, which was maintained within physiological range (37 ± 0.5°C) using a water-filled heating pad placed under the animal and an infrared lamp ∼50 cm over it. At the end of surgery, we injected an antibiotic (Cefovecin, 0.1 ml/kg) subcutaneously and taped a transdermal fentanyl patch (25 mcg/h) to the back of the animal 2–3 cm rostral to the base of the tail to provide prolonged analgesia (4- to 5-d period before removal). We also injected buprenorphine (0.01 mg/kg), a fast-acting analgesic, subcutaneously at the end of the surgery and a second dose ∼7 h later. Following surgery, we placed the cat in an incubator until they regained consciousness.

### Electrode implantation

We implanted all cats with electrodes to chronically record the electrical activity [electromyography (EMG)] of several forelimb and hindlimb muscles. We directed pairs of Teflon-insulated multistrain fine wires (AS633; Cooner Wire) subcutaneously from two head-mounted 34-pin connectors (Omnetics). Electrodes were sewn into the belly of selected forelimb and hindlimb muscles for bipolar recordings, with 1–2 mm of insulation stripped from each wire. We verified electrode placement during surgery by electrically stimulating each muscle through the matching head connector channel. The head connector was secured to the skull using dental acrylic and four to six metallic screws.

### Staggered hemisections and spinal transection

After collecting data in the intact state, a lateral hemisection was made between the fifth and sixth thoracic vertebrae on the right side of the spinal cord. General surgical procedures were the same as described above. The skin was incised between the fifth and sixth thoracic vertebrae and after carefully setting aside muscle and connective tissue, a small laminectomy of the dorsal bone was made. After exposing the spinal cord, we applied xylocaine (lidocaine hydrochloride, 2%) topically and made two to three injections on the right side of the cord. The right side of the spinal cord was then hemisected with surgical scissors between the fifth and sixth thoracic vertebrae. A hemostatic material (Spongostan) was inserted at the lesion site to stop residual bleeding, and muscles and skin were sewn back to close the opening in anatomic layers. In the days following hemisection, cats were carefully monitored for voluntary bodily functions by experienced personnel and bladder and large intestine were manually expressed as needed. The hindlimbs were cleaned as needed to prevent infection. After collecting data following the first hemisection, we performed a second lateral hemisection between the 10th and 11th thoracic vertebrae on the left side of the spinal cord 9–12 weeks later. Surgical procedures and postoperative care were the same as following the first hemisection. After the second hemisection, we collected data for 8–12 weeks. In three cats (TO, JA, HO), we performed a complete spinal transection at T12–T13 nine to 10 weeks after the second hemisection. We did not perform spinal transections in the other cats because we had to prematurely euthanize them at the start of the COVID-19 pandemic. Surgical procedures and postoperative care were the same as following the hemisections.

### Experimental protocol

We collected kinematic and EMG data before (intact state) and at four different time points before and after staggered hemisections during tied-belt (equal left-right speeds) quadrupedal locomotion at 0.4 m/s. The treadmill consisted of two independently controlled running surfaces 120 cm long and 30 cm wide (Bertec). A Plexiglas separator (120 cm long, 3 cm high, and 0.5 cm wide) was placed between the left and right belts to prevent the limbs from impeding each other. We present data collected at weeks 1–2 and 7–8 after the first and second hemisections. Cats were not trained to recover quadrupedal locomotion but data collection included several treadmill tasks, such as tied-belt locomotion from 0.4 to 1.0 m/s and split-belt locomotion (left slow/right fast and right slow/left fast), with both the right and left sides stepping on the slow and fast belts (Lecomte et al., 2022). Cats also performed overground locomotion in a straight line and in turns on a custom-built walkway, as well as obstacle negotiations (Lecomte et al., 2023). Some projects also included having cats walk on different surfaces (e.g., foam) to evaluate the influence of somatosensory feedback. We also evoked cutaneous reflexes in some cats by stimulating the superficial radial, superficial peroneal and distal tibial nerves during tied belt and split-belt locomotion at 0.4 and 0.8 m/s. In the intact state and after the first hemisection, nerves were also stimulated with longer trains to induce stumbling corrective reactions in the forelimbs and hindlimbs during treadmill locomotion at 0.4 and 0.8 m/s (Merlet et al., 2022). Other manuscripts are in preparation. In three cats, we collected data during hindlimb-only locomotion 1 d, 2 d, one week, two weeks, and three weeks after spinal transection with the forelimbs placed on a stationary platform. At two or three weeks after spinalization, we also collected data during quadrupedal treadmill locomotion at 0.4 m/s in these spinal cats.

In all locomotor trials and at all time points, the goal was to collect ∼15 consecutive cycles using positive reinforcement (food, affection). To avoid fatigue, ∼30 s of rest were given between trials. When required, an experimenter held the tail of the animal to provide mediolateral balance but not to provide weight support. In the double-hemisected and spinal states, some cats, required manual stimulation of the skin of the perineal region to facilitate hindlimb locomotion. For perineal stimulation, the same experimenter manually rubbed/pinched the perineal region with the index finger and thumb. As described, the strength of perineal stimulation is difficult to quantify but we adjusted the pressure applied to the perineal region on a case-by-case basis (light/strong, tonic/rhythmic) to achieve the best hindlimb locomotor pattern possible (Caron et al., 2020; Audet et al., 2022). Perineal stimulation increases spinal excitability and facilitates hindlimb locomotion in spinal mammals through an undefined mechanism (Merlet et al., 2021). However, if the perineal stimulation was too strong, we observed exaggerated flexion of the hindlimbs (hip, knee, and ankle) and/or improper left-right alternation, which impaired treadmill locomotion. In other words, too much excitability to spinal locomotor networks was detrimental.

### Data collection and analysis

We collected kinematic and EMG data as described previously (Harnie et al., 2018, 2019, 2021; Lecomte et al., 2021; Audet et al., 2022). Reflective markers were placed on the skin over bony landmarks: the scapula, minor tubercle of the humerus, elbow, wrist, metacarpophalangeal joint and at the tips of the toes for the forelimbs and over the iliac crest, greater trochanter, lateral malleolus, metatarsophalangeal joint and at the tip of the toes for the hindlimbs. Videos of the left and right sides were obtained with two cameras (Basler AcA640-100 g) at 60 frames/s with a spatial resolution of 640 × 480 pixels. A custom-made program (Labview) acquired the images and synchronized acquisition with EMG data. EMG signals were preamplified (10×, custom-made system), bandpass filtered (30–1000 Hz), and amplified (100–5000×) using a 16-channel amplifier (model 3500; A-M Systems). As we implanted >16 muscles per cat, we obtained data in each locomotor condition twice, one for each connector, as our data acquisition system is limited to 16 channels. EMG data were digitized (2000 Hz) with a National Instruments card (NI 6032E), acquired with custom-made acquisition software and stored on computer. In the present study, EMG data are used only for illustrative purposes to show the gait patterns before and after spinal lesions. Measures of EMG and more detailed descriptions will be presented in upcoming papers.

#### Temporal variables

By visual detection, the same experimenter determined, for all four limbs, paw contact as the first frame where the paw made visible contact with the treadmill surface, and liftoff as the most caudal displacement of the toes. We measured cycle duration from successive paw contacts, while stance duration corresponded to the interval of time from foot contact to the most caudal displacement of the toe relative to the hip/shoulder (Halbertsma, 1983). We calculated swing duration as cycle duration minus stance duration. Based on contacts and liftoffs for each limb, we measured individual periods of support (i.e., when two, three or four limbs are in contact with the treadmill surface), and expressed them as a percentage of cycle duration, as described previously (Frigon et al., 2014; Lecomte et al., 2022; Merlet et al., 2022). During a normalized cycle, here defined from successive right hindlimb contacts, we identified nine periods of limb support (Gray and Basmajian, 1968; Wetzel and Stuart, 1976; Frigon et al., 2014; Lecomte et al., 2022). We evaluated the coordination between the forelimbs and hindlimbs by measuring the phase interval between the right forelimb and hindlimb (right homolateral coupling; Thibaudier et al., 2017). The phase interval is the absolute amount of time between contact of the right hindlimb and right forelimb divided by the cycle duration of the right hindlimb (English, 1979; English and Lennard, 1982; Orsal et al., 1990; Frigon et al., 2014; Thibaudier and Frigon, 2014; Thibaudier et al., 2017; Audet et al., 2022). Because cats often perform 2:1 coordination (i.e., two right forelimb cycles for one right hindlimb cycle), we separated the first and second forelimb cycles when this occurred. Thus, in 2:1 fore-hind coordination patterns, there are two phase intervals for the first and second forelimb cycles. Phase interval values were then multiplied by 360 and expressed in degrees to illustrate their continuous nature and possible distributions (English and Lennard, 1982; Thibaudier et al., 2017).

#### Spatial variables

We analyzed spatial variables using DeepLabCut, an open-source machine learning program with deep neural network (Mathis et al., 2018), as we recently described in the cat (Lecomte et al., 2021). Stride length was measured for the right forelimbs and right hindlimbs as the distance between contact and liftoff added to the distance traveled by the treadmill during the swing phase, obtained by multiplying swing duration by treadmill speed (Courtine et al., 2005; Goetz et al., 2012; Thibaudier and Frigon, 2014; Dambreville et al., 2015; Lecomte et al., 2021). We measured the relative distance of the paw at contact and liftoff as the horizontal distance between the toe and shoulder or hip markers at stance onset and offset, respectively, for the right forelimbs and right hindlimbs. As an indicator of limb interference, we measured the horizontal distance between the toe markers of the forelimbs and hindlimbs on the same side at stance onset and offset of each of the four limbs of the animals.

### Histology and euthanasia

At the end of the experiments, cats were anesthetized with isoflurane before receiving a lethal dose (100 mg/kg) of pentobarbital through the left or right cephalic vein. The extent of the spinal lesion was confirmed by histology, as described previously (Lecomte et al., 2022, 2023). Following euthanasia, a 2-cm length of the spinal cord centered on the lesion sites was dissected and placed in 25 ml of 4% paraformaldehyde (PFA) solution (in 0.1 m PBS, 4°C). After 5 d, the spinal cord was cryoprotected in PBS with 30% sucrose for 72 h at 4°C. We then cut the spinal cord in 50 μm coronal sections on gelatinized slides using a cryostat (Leica CM1860, Leica Biosystems Inc). Sections were mounted on slides and stained with 1% cresyl violet. For staining, slides were then dehydrated in successive baths of ethanol 50%, 70%, and 100%, 5 min each. After a final 5 min in a xylene bath, slides were left to dry before being scanned by Nanozoomer (Hamamastu Corporation). We then performed qualitative and quantitative evaluations of the lesion sites in the transverse plane to estimate lesion extent.

### Statistical analysis

We performed statistical analyses using IBM SPSS Statistics 20.0 software. We first assessed the normality of each variable using the Shapiro–Wilk test. As the data were not parametric, we determined the effects of state/time points on dependent variables using the one-factor Friedman test for each state/time points. When a main effect was found, we performed a Wilcoxon signed-rank test with Bonferroni’s correction. The critical level for a statistical significance was set at an α-level of 0.05. Rayleigh’s test was performed to determine whether phase intervals were randomly distributed, as described (Zar, 1974; Kjaerulff and Kiehn, 1996; Thibaudier and Frigon, 2014; Thibaudier et al., 2017; Audet et al., 2022). Briefly, we calculated the *r* value to measure the dispersion of phase interval values around the mean, with a value of 1 indicating a perfect concentration in one direction, and a value of 0 indicating uniform dispersion. To test the significance of the directional mean, we performed Rayleigh’s z test: z = *nr*^2^, where *n* is the sample size (number of cycles). The z value was then compared with a critical z value on Rayleigh’s table to determine whether there was a significant concentration around the mean (*p* < 0.05).

## Results

### The recovery of quadrupedal treadmill locomotion after staggered hemisections and extent of spinal lesions

**Figure 1.**
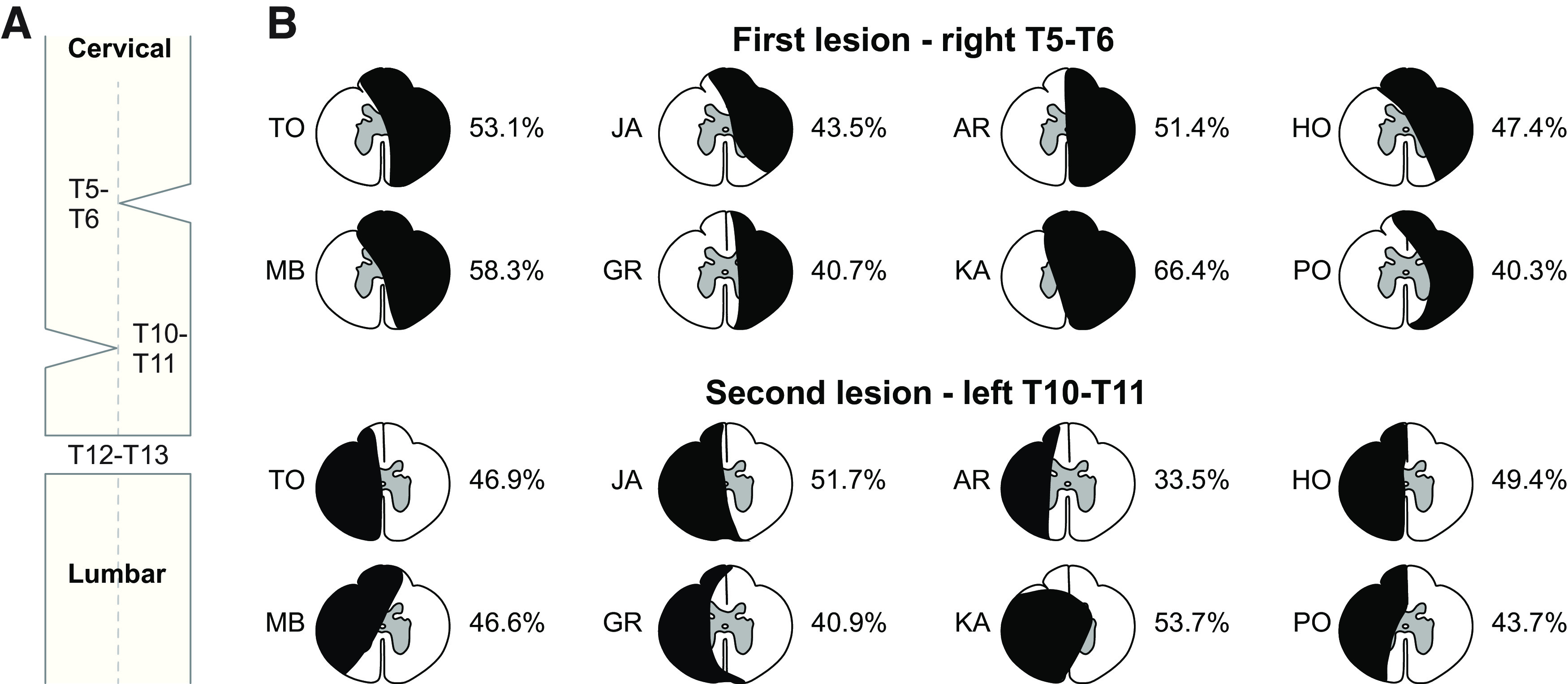
Staggered hemisections paradigm and extent of lesions. ***A***, Schematic representation of the staggered hemisections with the first and second hemisections at right (T5–T6) and left (T10–T11) thoracic levels, respectively, followed by a complete transection at T12–T13. ***B***, Estimation of first and second lesions extent based on histologic analyses for individual cats. The black area represents the lesioned region.

Figure 1 shows a schematic of the staggered hemisections (Fig. 1*A*) and an estimation of the extent of the first and second hemisections for each cat based on histologic analysis (Fig. 1*B*), which ranged from 40.3% to 66.4% (50.1 ± 9.1%) and 33.5% to 53.7% (45.8 ± 6.5%) for the first and second lesions, respectively. In the present study, all eight cats spontaneously recovered quadrupedal treadmill locomotion at 0.4 m/s one to two weeks following the first lateral hemisection at T5–T6 on the right side. All eight cats also recovered quadrupedal treadmill locomotion at 0.4 m/s one to four weeks following the second lateral hemisection at T10–T11 on the left side. Thus, in some cats, the first time point after the second hemisection was at two, three or four weeks (see Table 1).

**Table 1:** Locomotor performance of individual cats after the first and second hemisections

Cat ID	Time point	Balance assistance	Perineal stimulation required	Left digitigrade paw placement	Right digitigrade paw placement
TO	Hemi 1, wk 2 Hemi 1, wk 8 Hemi 2, wk 3 Hemi 2, wk 7	No No Yes Yes	No No Yes No	Yes Yes Yes Yes	Yes Yes Yes Yes
JA	Hemi 1, wk 2 Hemi 1, wk 8 Hemi 2, wk 2 Hemi 2, wk 7	No No Yes Yes	No No Yes No	Yes Yes Yes Yes	Yes Yes Yes Yes
AR	Hemi 1, wk 2 Hemi 1, wk 8 Hemi 2, wk 1 Hemi 2, wk 7	Yes No Yes Yes	Yes No Yes Yes	Yes Yes Yes Yes	Yes Yes Yes Yes
HO	Hemi 1, wk 2 Hemi 1, wk 8 Hemi 2, wk 3 Hemi 2, wk 7	No No Yes Yes	No No No No	Yes Yes Yes Yes	Yes Yes Yes Yes
MB	Hemi 1, wk 2 Hemi 1, wk 7 Hemi 2, wk 3 Hemi 2, wk 8	No No Yes Yes	No No Yes Yes	Yes Yes No No	Yes Yes 71% 48%
GR	Hemi 1, wk 1 Hemi 1, wk 8 Hemi 2, wk 3 Hemi 2, wk 8	No¸ No Yes Yes	No No No No	Yes Yes 57% Yes	70% Yes Yes Yes
KA	Hemi 1, wk 2 Hemi 1, wk 8 Hemi 2, wk 3 Hemi 2, wk 7	No No Yes Yes	No No No No	Yes Yes Yes Yes	Yes Yes Yes Yes
PO	Hemi 1, wk 1 Hemi 1, wk 8 Hemi 2, wk 4 Hemi 2, wk 7	No No Yes Yes	No No Yes Yes	Yes Yes 26% Yes	No Yes Yes Yes

Locomotor performance of eight cats using four criteria (balance assistance, requirement of perineal stimulation to evoke locomotion, and proper digitigrade placement of the left and right hindpaw). % values indicate the percentage of cycles with correct digitigrade placement in some cats. The left column is cat identification (ID). In the time point column, Hemi 1 and Hemi 2 refer to the first and second hemisections, respectively. The week that data were collected after the first and second hemisections is indicated for each time point in individual cats. wk = week.

Table 1 summarizes three features of locomotor performance after the first and second hemisections. After the first hemisection, most cats (seven of eight) did not require balance assistance (experimenter holds the tail to provide mediolateral balance but not weight support). Cats did not require perineal stimulation to perform quadrupedal locomotion after the first hemisection. After the second hemisection, all cats required balance assistance at both time points and five of eight and three of eight cats required perineal stimulation at weeks 1–4 and 7–8, respectively. The three cats requiring perineal stimulation at weeks 7–8 also needed it at weeks 1–4.

After the first hemisection on the right side, all eight cats maintained left digitigrade hindpaw placement (contralesional). Most cats (six of eight) also retained right digitigrade hindpaw placement (ipsilesional). At weeks 7–8 after the first hemisection, all cats performed left and right digitigrade placement. The second hemisection on the left side did not affect digitigrade placement of the right hindpaw in seven of eight cats. Surprisingly, most cats (five out of eight) maintained left digitigrade hindpaw placement at weeks 1–4 after the second hemisection on the left side.

### New patterns of forelimb-hindlimb coordination emerge after the first and second hemisections

**Figure 2.**
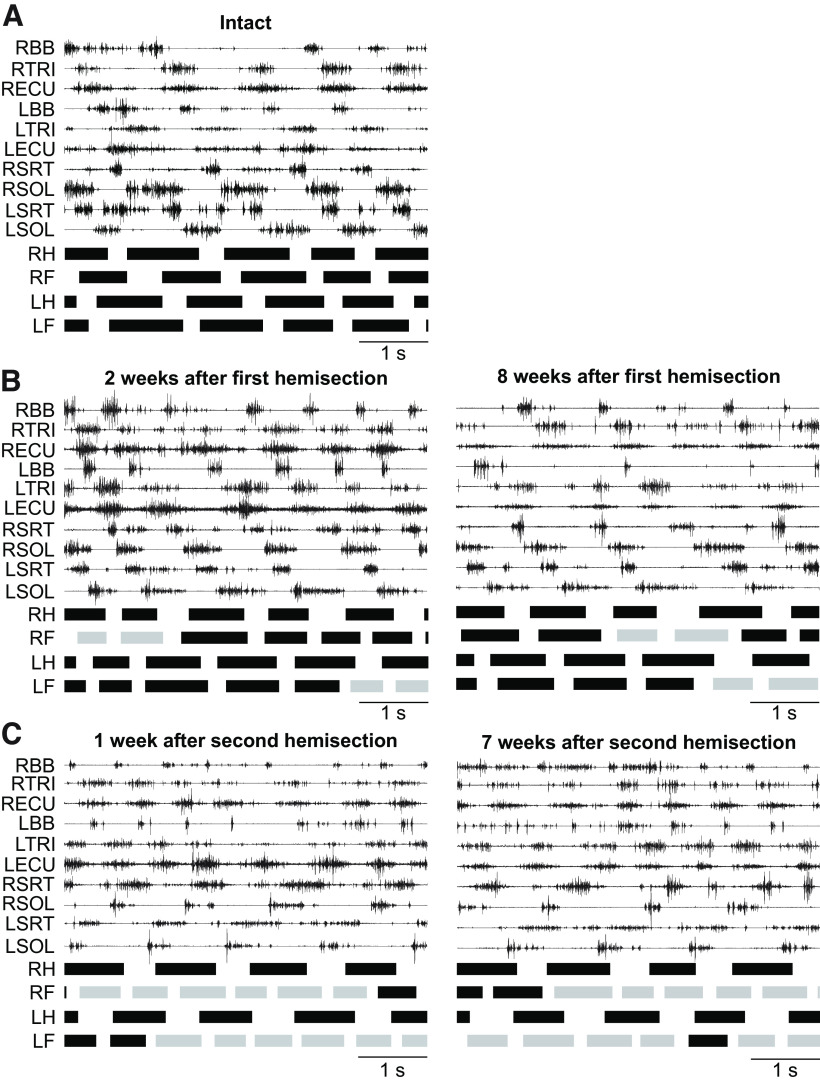
Quadrupedal treadmill locomotion before and after staggered hemisections. Electromyography from selected forelimb and hindlimb muscles along with stance phases (thick horizontal lines) of the left (L) and right (R) limbs in Cat AR at 0.4 m/s in (***A***) the intact state, (***B***) after the first hemisection, and (***C***) after the second hemisection. Gray stance phases indicate cycles with 2:1 fore-hind coordination. Note two cycles of the left (LF) or right (RF) forelimb within one right hindlimb (RH) cycle. BB, Biceps brachii; LH, left hindlimb; TRI, Triceps brachii; ECU, extensor carpi ulnaris; SRT, sartorius; SOL, soleus.

In the present study, all intact cats performed 1:1 fore-hind coordination in 100% of trials, indicating an equal number of forelimb and hindlimb cycles, as shown for a single intact cat in Figure 2*A*. However, after the first and second hemisections, all cats showed 2:1 fore-hind coordination with varying proportions, where the left or right forelimbs performed two cycles within one right hindlimb cycle. When this occurred, cycles with 2:1 and 1:1 fore-hind coordination were intermingled within the same locomotor episode (Fig. 2*B*,*C*) and some cats only showed patterns of 2:1 fore-hind coordination (100%). The appearance of 2:1 fore-hind coordination as been reported in several studies rats and cats (Górska et al., 1990, 1996, 2013; Bem et al., 1995; Barrière et al., 2010; Alluin et al., 2011; Leszczyńska et al., 2015; Thibaudier et al., 2017). Table 2 summarizes the proportion of 2:1 fore-hind coordination in each cat after both hemisections.

**Table 2:** Proportion of 2:1 fore-hind coordination after the first and second hemisections

	First hemisection		Second hemisection	
Cat ID	Weeks 1–2	Weeks 7–8	Weeks 1–4	Weeks 7–8
TO	100% (14/14)	100% (16/16)	62% (8/13)	100% (12/12)
JA	58% (7/12)	58% (11/19)	100% (8/8)	96% (22/23)
AR	50% (4/8)	100% (21/21)	75% (18/24)	96% (23/24)
HO	25% (6/24)	20% (2/10)	71% (12/17)	71% (15/21)
MB	100% (22/22)	82% (28/34)	29% (4/14)	11% (2/19)
GR	10% (1/10)	19% (7/36)	52% (11/21)	50% (6/12)
KA	15% (4/26)	9% (2/23)	100% (21/21)	71% (12/17)
PO	33% (6/18)	13% (1/8)	89% (17/19)	88% (7/8)

Percent values indicate the percentage of cycles with 2:1 fore-hind coordination, where the left or right forelimb performed two cycles within a right hindlimb cycle. The number in brackets indicates the number of cycles with 2:1 fore-hind coordination dived by the total number of hindlimb cycles recorded for individual cats after the first and second hemisections. The left column is cat identification (ID).

### Interlimb coordination is weaker and more variable after staggered hemisections

To determine how the first and second hemisections affected the coordination between the forelimbs and hindlimbs, we measured phase intervals between the right forelimb and right hindlimb (right homolateral coupling). We separated 1:1 and 2:1 fore-hind coordination patterns. Thus, in 2:1 coordination, there are two phase interval values, one for the first and second right forelimb cycles within a right hindlimb cycle. We only show this analysis for right homolateral coupling because the other couplings between the forelimbs and hindlimbs (left homolateral and both diagonal couplings) are equivalent.

**Figure 3.**
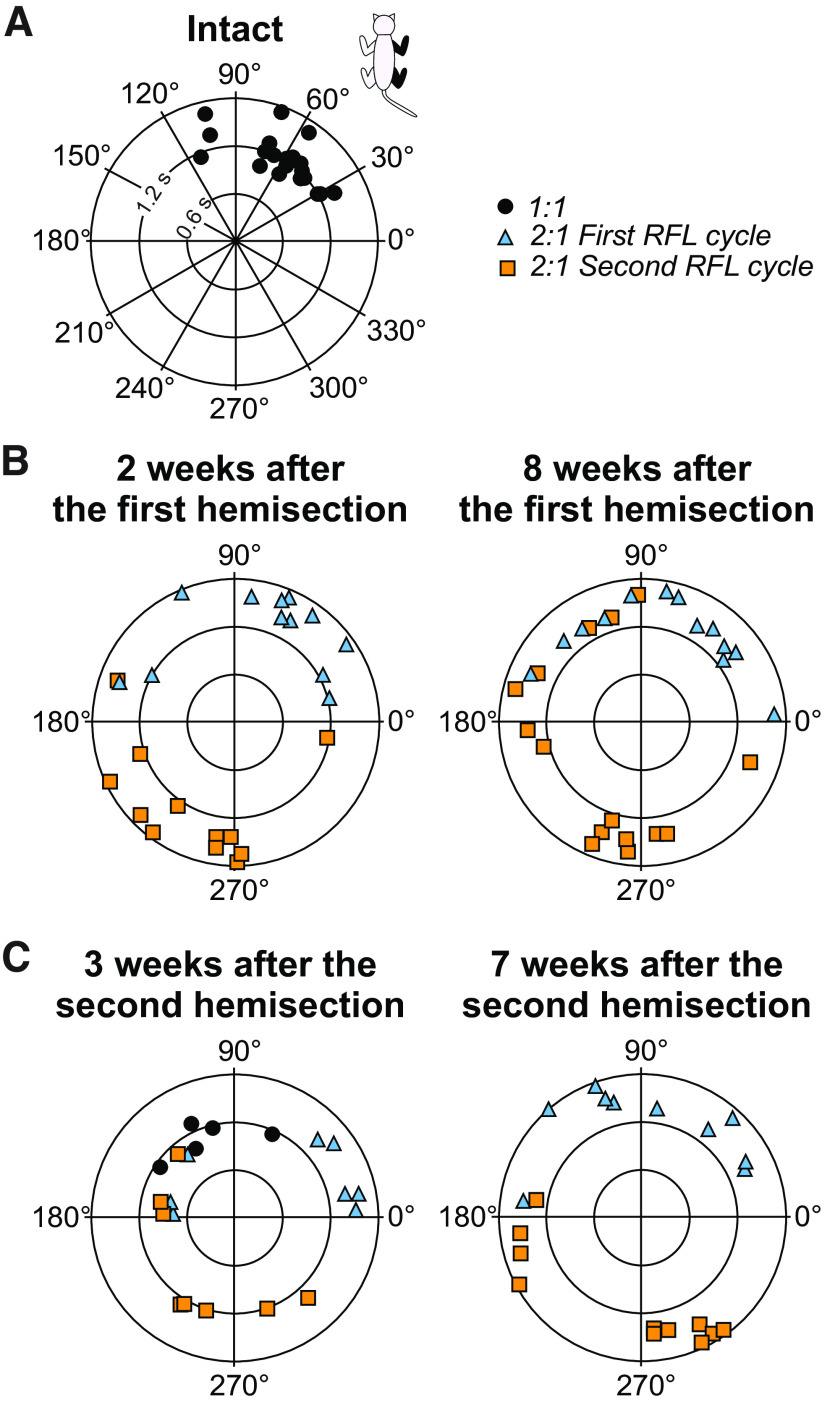
Coordination between right homolateral limbs before and after staggered hemisection. Polar plots showing phase interval values for right homolateral coupling (phasing between right forelimb (RFL) and right hindlimb contacts) in degrees around the circumference in (***A***) the intact state, (***B***) after the first hemisection, and (***C***) after the second hemisection. After the first and second hemisection, we separated cycles with 1:1 and 2:1 fore-hind coordination where RFL performs two cycles within one right hindlimb cycle. Thus, two phase interval values are found in 2:1 coordination for the first and second forelimb cycles. Right hindlimb cycle durations increase from the center and are plotted in radii. Data are from cat TO.

Figure 3 shows an example of right homolateral coupling phase intervals from a single cat (Cat TO). As can be seen, in the intact state, all phase interval values were clustered between 30° and 120°, and only 1:1 fore-hind coordination was observed (Fig. 3*A*). After the first hemisection, only 2:1 coordination was present in this cat and phase interval values for the first and second forelimb cycles were more dispersed than in the intact state (Fig. 3*B*). At week 3 after the second hemisection, cycles with 1:1 coordination returned and were intermingled with 2:1 coordination (Fig. 3*C*). Phase interval values remained dispersed. At week 7 after the second hemisection, only 2:1 coordination was observed and phase interval values remained dispersed.

To assess the step-by-step consistency of right homolateral coupling, we performed Rayleigh’s test and calculated the *r* value, a measure of angular dispersion around the mean. When the *r* value is close to 1.0 and significant, it indicates that phase intervals are oriented in a specific direction. Table 3 shows *r* values from Rayleigh’s test for right homolateral couplings for individual cats where we separated cycles with 1:1 and 2:1 fore-hind coordination. Note that some cats did not display 1:1 coordination after the first and/or second hemisections. All cats had 1:1 coordination in the intact state, with *r* values ranging from 0.79 to 1.00 (mean 0.92 ± 0.07), indicating consistent step-by-step fore-hind coordination. At weeks 1–2 after the first hemisection, six of eight cats had cycles with 1:1 coordination, with *r* values ranging from 0.45 to 0.94 (mean 0.78 ± 0.21). At weeks 1–2 after the first hemisection, seven of eight cats had cycles with 2:1 coordination, with *r* values for the first forelimb cycle ranging from 0.12 to 0.85 (mean 0.48 ± 0.29). For the second forelimb cycle, *r* values ranged from 0.27 to 0.70 (mean 0.54 ± 0.15). At weeks 7–8 after the first hemisection, six of eight cats had cycles with 1:1 coordination, with *r* values ranging from 0.13 to 0.85 (mean 0.61 ± 0.26). Six of eight cats had cycles with 2:1 coordination, with *r* values ranging from 0.30 to 0.84 (mean 0.50 ± 0.23) and 0.12 to 0.80 (mean 0.40 ± 0.25) for the first and second forelimb cycles, respectively. At weeks 1–4 after the second hemisection, six of eight cats had cycles with 1:1 coordination, with *r* values ranging from 0.38 to 0.91 (mean 0.62 ± 0.22). All eight cats had cycles with 2:1 coordination, with *r* values ranging from 0.39 to 0.83 (mean 0.51 ± 0.14) and 0.12 to 0.86 (mean 0.49 ± 0.21) for the first and second forelimb cycles, respectively. At weeks 7–8 after the second hemisection, only four of eight cats had cycles with 1:1 coordination, with *r* values ranging from 0.44 to 0.97 (mean 0.72 ± 0.24). All eight cats had cycles with 2:1 coordination, with *r* values ranging from 0.35 to 0.70 (mean 0.49 ± 0.11) and 0.10 to 0.64 (mean 0.43 ± 0.17) for the first and second forelimb cycles, respectively.

**Table 3:** Circular statistics for forelimb-hindlimb coordination before and after staggered hemisections

		1:1 coordination	2:1, 1st forelimb cycle	2:1, 2nd forelimb cycle
Cat ID	Time points	*r*	*r*	*r*
TO	Intact	0.79*	-	-
	Hemi 1, wk 2	-	0.74*	0.68*
	Hemi 1, wk 8	-	0.75*	0.44*
	Hemi 2, wk 3	0.91*	0.42	0.60*
	Hemi 2, wk 7	-	0.70*	0.64*
JA	Intact	0.86*	-	-
	Hemi 1, wk 2	0.45	0.64	0.27
	Hemi 1, wk 8	0.74*	0.30	0.35
	Hemi 2, wk 2	-	0.83*	0.86*
	Hemi 2, wk 6	-	0.58*	0.33
AR	Intact	0.88*	-	-
	Hemi 1, wk 2	0.94*	0.85	0.49
	Hemi 1, wk 8	-	0.84*	0.80*
	Hemi 2, wk 1	0.80*	0.47*	0.54*
	Hemi 2, wk 7	-	0.51*	0.57*
HO	Intact	0.97*	-	-
	Hemi 1, wk 2	0.57*	0.23	0.48
	Hemi 1, wk 8	0.69*	-	-
	Hemi 2, wk 3	0.44	0.46	0.48
	Hemi 2, wk 7	0.87*	0.42	0.42
MB	Intact	0.96*	-	-
	Hemi 1, wk 2	-	0.23	0.54*
	Hemi 1, wk 7	0.52	0.33	0.50*
	Hemi 2, wk 3	0.71*	0.39	0.49
	Hemi 2, wk 8	0.60*	0.48	0.10
GR	Intact	1.00*	-	-
	Hemi 1, wk 1	0.92*	-	-
	Hemi 1, wk 8	0.75*	0.37	0.17
	Hemi 2, wk 3	0.38	0.61*	0.12
	Hemi 2, wk 8	0.44	0.41	0.39
KA	Intact	0.97*	-	-
	Hemi 1, wk 2	0.93*	0.12	0.64
	Hemi 1, wk 8	0.85*	0.42	0.12
	Hemi 2, wk 3	-	0.46*	0.38
	Hemi 2, wk 7	0.97*	0.48	0.57*
PO	Intact	0.90*	-	-
	Hemi 1, wk 1	0.88*	0.55	0.70*
	Hemi 1, wk 8	0.13	-	-
	Hemi 2, wk 4	0.46	0.46*	0.47*
	Hemi 2, wk 7	-	0.35	0.43

The table shows *r* values from Rayleigh’s test at the different time points for individual cats before and after hemisections for cycles with 1:1 and 2:1 (first and second forelimb cycles) coordination for right homolateral coupling (the phasing between right forelimb and right hindlimb contacts). The *r* value measures the dispersion of phase interval values around the mean, with a value of 1 indicating a perfect concentration in one direction and a value of 0 indicating uniform dispersion. We performed Rayleigh’s z test: z = *nr*^2^, where *n* is the sample size (number of cycles) and compared the z value to a critical z value on Rayleigh’s table to determine whether there was a significant concentration around the mean (*p* < 0.05). Asterisks indicate a significant *r* value. The left column is cat identification (ID). wk = week.

### Staggered hemisections generate temporal adjustments in the forelimbs and hindlimbs and reversals of left-right asymmetries in the hindlimbs

**Figure 4.**
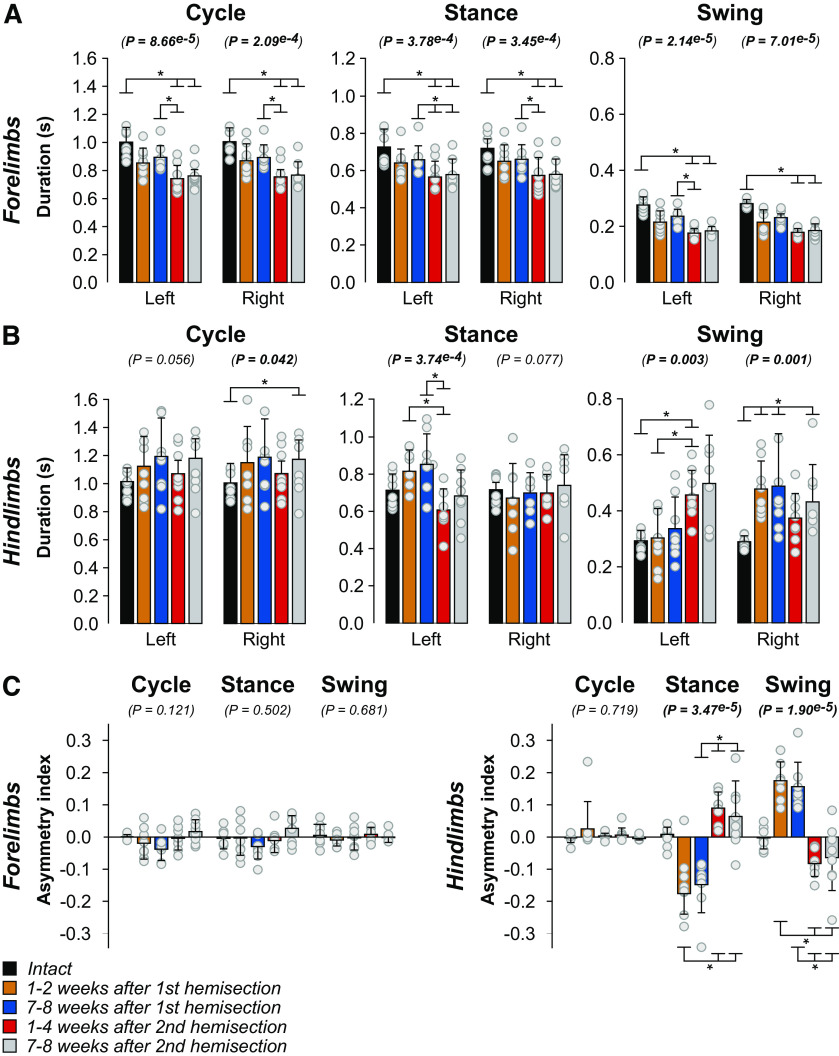
Temporal adjustments during quadrupedal treadmill locomotion before and after staggered hemisections for the group. ***A***, ***B***, Cycle, stance and swing durations for the forelimbs and hindlimbs, respectively. ***C***, Asymmetry indexes of temporal variables (right limb durations minus left limb durations). We averaged 8–36 cycles per cat. The bars represent the mean ± SD for the group (*n* = 8 cats) while gray circles represent individual data points (mean for each cat). The *p* values show the main effect of state (one-factor Friedman test). Asterisks indicate significant differences between time points from the Wilcoxon signed-rank test with Bonferroni’s correction.

To determine temporal adjustments of the forelimbs and hindlimbs during quadrupedal treadmill locomotion, we measured cycle and phase durations before and after the two hemisections. We pooled cycles with 1:1 and 2:1 fore-hind coordination because some cats did not show 1:1 coordination after the first and/or second hemisections. For the forelimbs (Fig. 4*A*), we observed a significant reduction in left (LF) and right (RF) forelimb cycle and stance durations after the second hemisection at weeks 1–2 and 7–8 compared with the intact state and at weeks 1–4 after the second hemisection compared with weeks 7–8 after the first (Fig. 4*A*). Compared with the intact state, LF and RF swing durations were significantly reduced at weeks 1–4 and 7–8 after the second hemisection, and at weeks 1–4 after the second hemisection compared with weeks 7–8 after the first for LF. Changes in forelimb cycle and phase durations are undoubtedly because of the appearance of 2:1 fore-hind coordination (see Table 2).

For the hindlimbs (Fig. 4*B*), we observed no significant change in left hindlimb (LH) cycle duration after staggered hemisections, but we found a main effect for right hindlimb (RH) cycle duration with an increase at weeks 7–8 after the second hemisection compared with the intact state. LH stance duration did not change significantly compared with the intact state after staggered hemisections, but we did observe a significant decrease at weeks 1–4 after the second hemisection compared with weeks 1–2 and 7–8 after the first. RH stance duration did not change significantly after staggered hemisections. LH swing duration was longer at weeks 1–4 after the second hemisection compared with the intact state and weeks 1–2 after the first hemisection. RH swing duration was longer at weeks 1–2 and 7–8 after the first hemisection and at weeks 7–8 after the second hemisection compared with the intact state.

To determine whether staggered hemisections produced left-right asymmetries in cycle and phase durations at shoulder and hip girdles, we measured an asymmetry index by subtracting right limb durations from left limb durations (Fig. 4*C*). We found no significant asymmetries in the forelimbs. However, for the hindlimbs, while we observed no asymmetries in cycle duration (cats maintained 1:1 coordination between hindlimbs), The asymmetry index for hindlimb stance duration became negative after the first hemisection (LH stance > RH stance), before switching to positive after the second hemisection (RH stance > LH stance). This suggests that weight support of the hindquarters shifted to the left contralesional side after the first hemisection and to the right side after the second hemisection of the left spinal cord. The asymmetry index for hindlimb swing durations showed an opposite pattern, becoming positive (RH swing > LH swing) and negative (LH swing > RH swing) after the first and second hemisections, respectively.

### Cats adjust their support periods after staggered hemisections during quadrupedal locomotion

**Figure 5.**
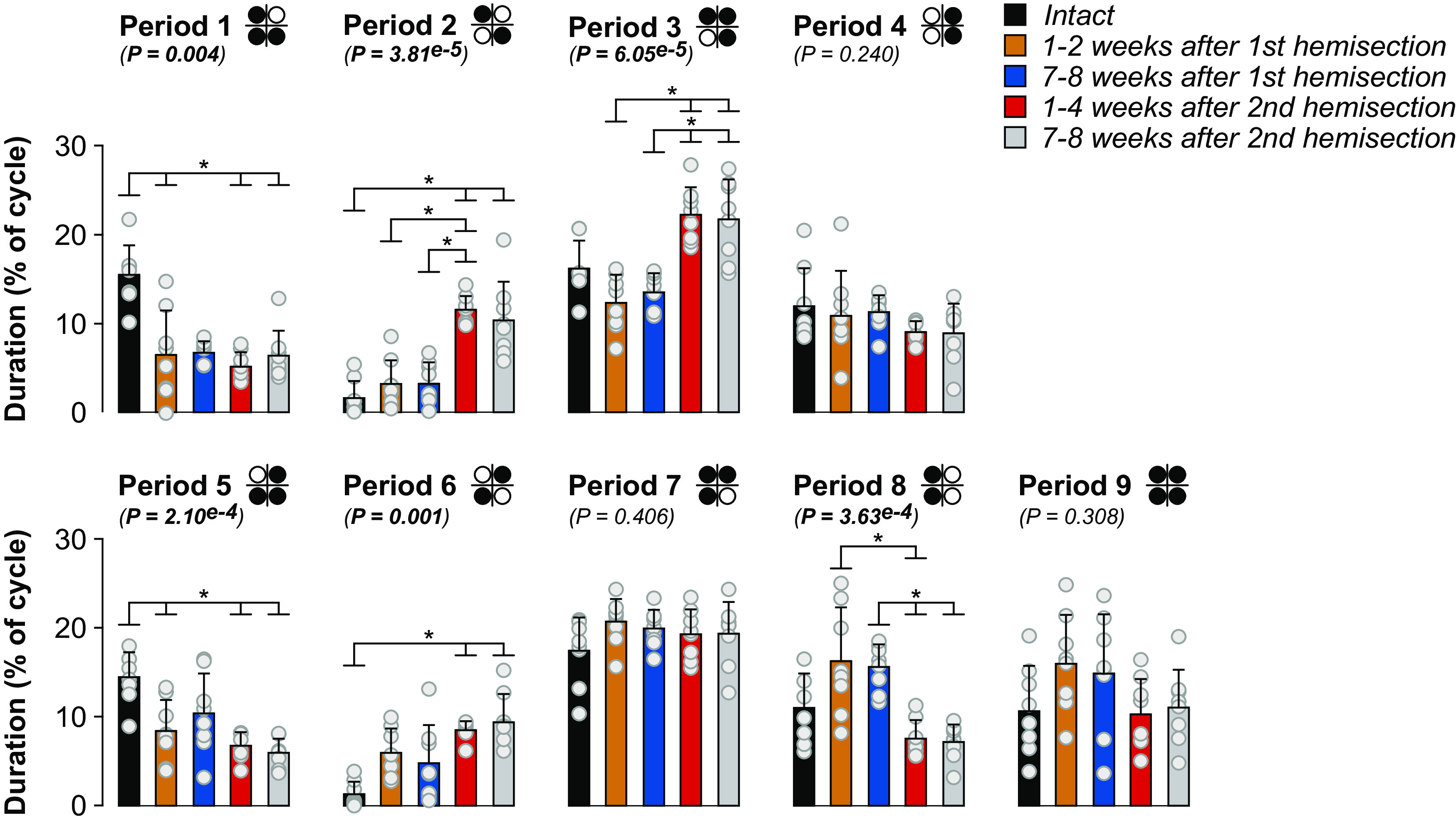
Support periods during quadrupedal treadmill locomotion before and after staggered hemisections for the group. Individual periods of support (nine in total) normalized to right hindlimb cycle duration. The diagrams show the limbs contacting the treadmill surface (in black) while open/white circles indicate limbs in an aerial phase. We averaged 8–36 cycles per cat. The bars represent the mean ± SD for the group (*n* = 8 cats) while gray circles represent individual data points (mean for each cat). The *p* values show the main effect of state (one-factor Friedman test). Asterisks indicate significant differences between time points from the Wilcoxon signed-rank test with Bonferroni’s correction.

We generally find eight individual support periods during quadrupedal locomotion in a normalized cycle (Frigon et al., 2014; Lecomte et al., 2022), as shown in order of occurrence in a locomotor cycle in Figure 5. A period of double support can become a period of quadrupedal support in some cycles; thus, we can find nine different support periods. The proportion of some support periods significantly increased after spinal hemisections, while others decreased (Fig. 5). For example, the two periods of triple support involving both hindlimbs (Periods 1 and 5) decreased after the two hemisections compared with the intact state, except at weeks 7–8 after the first hemisection. Periods of diagonal support (Periods 2 and 6) increased after the second hemisection compared with the intact state. Period 2, involving the left forelimb and right hindlimb, increased significantly at weeks 1–4 and 7–8 after the second hemisection compared with the intact state and at weeks 1–4 after the second compared with weeks 1–2 and 7–8 after the first. Period 6 increased at weeks 1–4 and 7–8 after the second hemisection compared with the intact state. The triple support period involving the two forelimbs and the right hindlimb (Period 3) increased after the second hemisection at weeks 1–4 and 7–8 compared with weeks 1–2 and 7–8 after the first. Left homolateral double support (Period 8) did not change significantly after staggered hemisections compared with the intact state. However, it was significantly shorter at weeks 1–4 after the second hemisection compared with both time points after the first hemisection and at weeks 7–8 after the second compared with weeks 7–8 after the first. We observed no significant changes after staggered hemisections for right homolateral support (Period 4), the triple support period involving the left hindlimb and both forelimbs (Period 7) and quadrupedal support (Period 9). Therefore, cats adjust their support periods to maintain dynamic balance during quadrupedal locomotion after staggered hemisections, mainly by decreasing periods of triple support involving both hindlimbs and one forelimb early after the first hemisection (on the right side), and then away from the left hindlimb after the second hemisection (on the left side).

### Staggered hemisections generate spatial adjustments in the forelimbs and hindlimbs but few left-right spatial asymmetries in the hindlimbs

**Figure 6.**
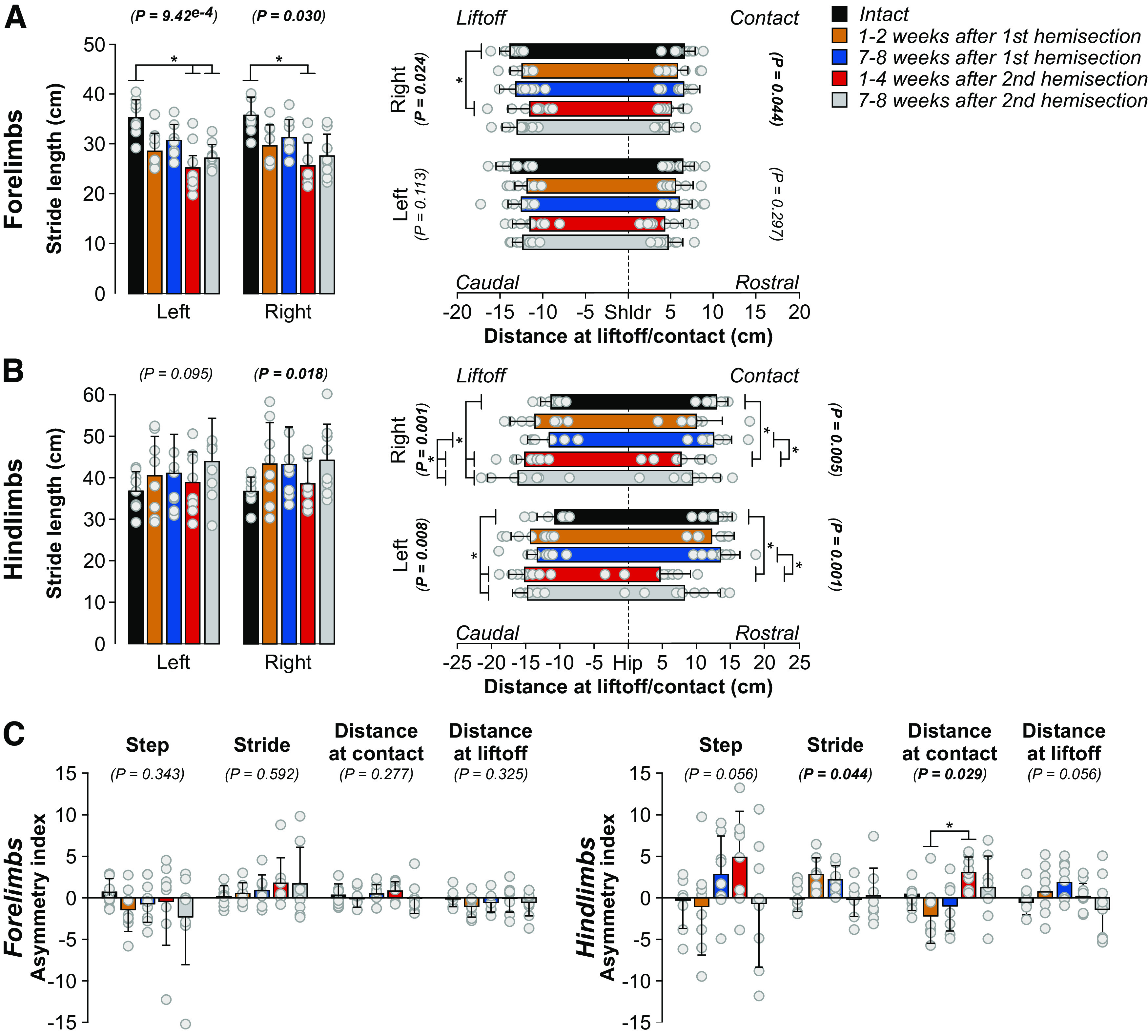
Spatial adjustments during quadrupedal treadmill locomotion before and after staggered hemisections for the group. ***A***, ***B***, Stride length and distances at contact and liftoff for the forelimbs and hindlimbs, respectively. ***C***, Asymmetry indexes of spatial variables (right limb values minus left limb values). We averaged 8–36 cycles per cat. The bars represent the mean ± SD for the group (*n* = 8 cats) while gray circles represent individual data points (mean for each cat). The *p* values show the main effect of state (one-factor Friedman test). Asterisks indicate significant differences between time points from the Wilcoxon signed-rank test with Bonferroni’s correction.

To determine how staggered hemisections affected spatial parameters, we measured stride length, the horizontal distance traveled by each limb from contact to contact and the horizontal distance of the forepaws and hindpaws from the shoulder and hip, respectively, at contact and liftoff. Compared with the intact state, forelimb stride lengths decreased bilaterally but only after the second hemisection, consistent with smaller steps with 2:1 fore-hind coordination (Fig. 6*A*). We observed that the distance of RF relative to the shoulder at liftoff was more rostral at weeks 1–4 after the second hemisection compared with the intact state while LF positioning did not change. Forelimb placement at contact relative to the shoulder did not change significantly.

Hindlimb stride length did not significantly change after staggered hemisections for LH and although RH showed a significant main effect, we observed no significant difference between time points (Fig. 6*B*). However, we observed several changes in the position of the hindpaw relative to the hip. We found a more caudal horizontal distance between the left hindpaw and the hip at liftoff at both time points after the second hemisection compared with the intact state. Similarly, we found a more caudal horizontal distance between the right hindpaw and the hip at liftoff at both time points after the second hemisection compared with the intact state and at weeks 7–8 after the first hemisection. The right and left hindpaw were closer to the hip at contact at weeks 1–4 after the second hemisection compared with the intact state and weeks 7–8 after the first hemisection.

To determine whether staggered hemisections produced asymmetric changes in spatial variables between the left and right sides at shoulder and hip girdles, we measured an asymmetry index by subtracting right limb values from left limb values (Fig. 6*C*). For the forelimbs, we found no significant asymmetries. For the hindlimbs, we found a significant main effect for stride length but pairwise comparisons revealed no differences between time points. For the distance at contact, we only observed a significant difference between weeks 1–2 after the first hemisection and weeks 1–4 after the second hemisection, where left and right placements were more rostral relative to the hip after the first and second hemisections, respectively.

### Forelimb movements adjust to avoid interference after staggered hemisections

**Figure 7.**
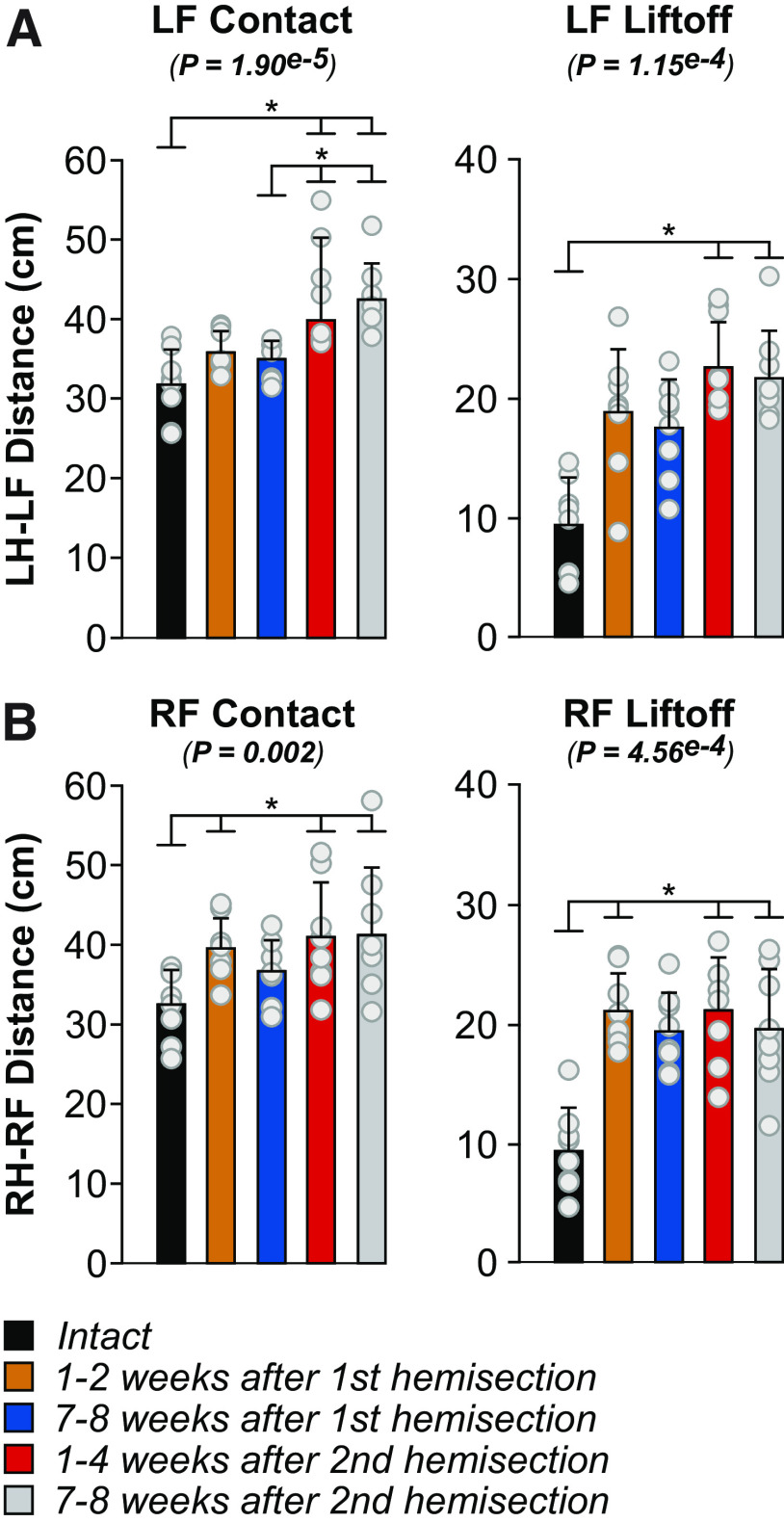
Homolateral limb interference during quadrupedal treadmill locomotion before and after staggered hemisections for the group. Each panel shows horizontal distances between homolateral limbs at contact and liftoff of (***A***) the left (LF) and (***B***) right (RH) forelimb. We averaged 8–36 (17.94 ± 7.08) cycles per cat. The bars represent the mean ± SD for the group (*n* = 8 cats), while gray circles represent individual data points (mean for each cat). The *p* values show the main effect of state (one-factor Friedman test). Asterisks indicate significant differences between time points from the Wilcoxon signed-rank test with Bonferroni’s correction. LH, left hindlimb; RH, right hindlimb.

To assess limb interference, we measured the horizontal distance between the toe markers of the forelimbs and hindlimbs at contact and liftoff of the left and right forelimbs (Fig. 7), as described previously in spinal cats during quadrupedal locomotion (Audet et al., 2022). The left distance, the distance between LF and LH toe markers, increased at LF contact at weeks 1–4 and 7–8 after the second hemisection compared with the intact state and compared with weeks 7–8 after the first hemisection (Fig. 7*A*, left panel). At LF liftoff, the left distance increased at weeks 1–4 and 7–8 after the second hemisection compared with the intact state (Fig. 7*A*, right panel). The right distance, the distance between RF and RH toe markers, increased at weeks 1–2 after the first hemisection and at weeks 1–4 and 7–8 after the second hemisection at both RF contact (Fig. 7*B*, left panel) and liftoff (Fig. 7*D*, right panel) compared with the intact state. We propose that increased distances between the forelimbs and hindlimbs helps avoid interference between the forelimbs and hindlimbs. The increase in distance between the right limbs after the second hemisection could also improve balance by increasing the support polygon.

### The recovery of hindlimb locomotion after staggered hemisections is mediated by a spinal mechanism

**Figure 8.**
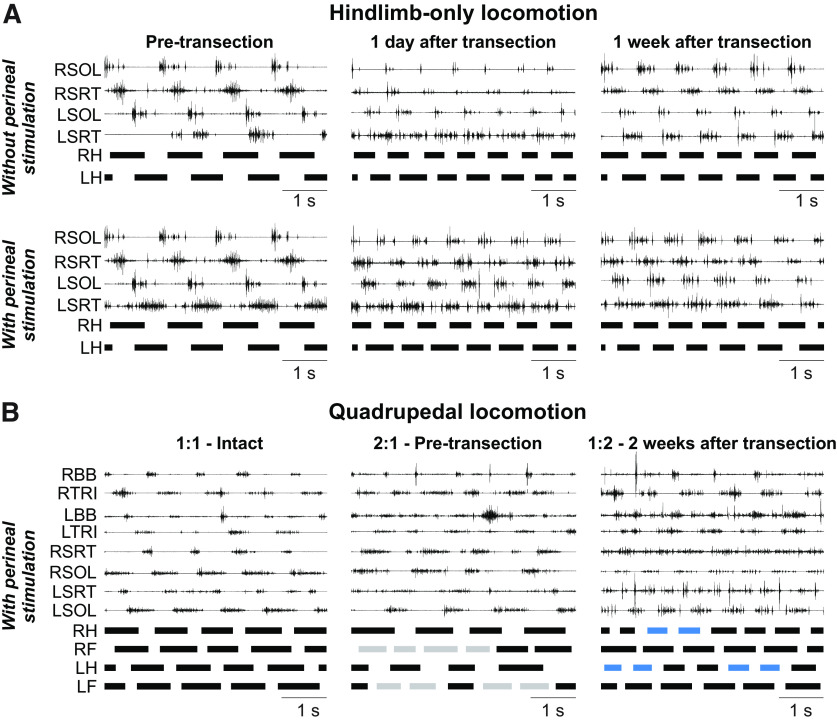
Hindlimb-only and quadrupedal treadmill locomotion before and after complete spinal transection. ***A***, Activity from selected hindlimb muscles and stance phases (thick horizontal lines) of the left (LH) and right (RH) hindlimbs in Cat JA at 0.4 m/s. ***B***, Activity from selected hindlimb muscles and stance phases (thick horizontal lines) of the left and right limbs in Cat HO at 0.4 m/s. Gray and blue stance phases indicate cycles with 2:1 (two forelimb cycles within one right hindlimb cycle) and 1:2 (two hindlimb cycles within one right forelimb cycle) fore-hind coordination, respectively. BB, Biceps brachii; LH, left hindlimb; RH, right hindlimb; SOL, soleus; SRT, sartorius; TRI, triceps brachii.

As stated in the introduction, a spinal mechanism plays a prominent role in the recovery of hindlimb locomotion following an incomplete SCI (Barrière et al., 2008). To determine whether a spinal mechanism also contributes to hindlimb locomotor recovery after staggered hemisections, we performed a spinal transection at T12–T13 9–10 weeks after the second hemisection in three cats (TO, HO, JA). In all three cats, hindlimb locomotion was expressed the day following transection, a recovery that normally takes a minimum of three weeks (Lovely et al., 1986; Barbeau and Rossignol, 1987; Barrière et al., 2008; Harnie et al., 2019). Figure 8*A* shows a representative example from one cat before transection (i.e., data collected at week 7 after the second hemisection), and at 1 d and one week following transection without (top panel) and with (bottom panel) perineal stimulation. Cat JA stepped 1 d after the transection without and with perineal stimulation. The pattern was maintained one week after transection.

We and others have shown that cats can perform quadrupedal locomotion after a complete thoracic spinal transection (Shurrager and Dykman, 1951; Eidelberg et al., 1980; Howland et al., 1995; Audet et al., 2022). In the present study, all three cats performed quadrupedal treadmill locomotion at 0.4 m/s with perineal stimulation and balance assistance after spinal transection. Instead of 1:1 and 2:1 fore-hind coordination patterns observed at weeks 7–8 after the second hemisection, we observed a 1:2 fore-hind coordination in some cycles, indicating that one hindlimb performed two cycles within a single forelimb cycle (Fig. 8*B*), as shown recently in spinal-transected cats (Audet et al., 2022). In all three cats, cycles with 1:2 fore-hind coordination were interspersed with 1:1 coordination. The 1:2 fore-hind coordination represented 21% (Cat TO), 20% (Cat JA) and 48% (Cat HO) of cycles. It is possible that perineal stimulation played a role in the emergence of 1:2 coordination.

## Discussion

We showed that cats spontaneously recovered quadrupedal locomotion following staggered hemisections but required balance assistance after the second. We hypothesized that the second hemisection would more greatly disrupt fore-hind coordination. Although the second hemisection did not change the step-by phasing of the forelimbs and hindlimbs compared with after the first hemisection, we observed changes in some support periods and some increases in the distance between the forelimbs and hindlimbs, consistent with an additional effect of the second hemisection on fore-hind coordination. Consistent with our hypothesis, hindlimb locomotion was expressed the day after spinal transection in cats that had recovered following the second hemisection. Below we discuss adjustments in the pattern and potential neuroplastic changes that allowed cats to maintain and recover some level of quadrupedal locomotor functionality.

### Recovery of posture and locomotion after staggered hemisections

Lesion extent varied between animals (Fig. 1). Generally, smaller lesions associate with faster and more complete locomotor recovery (Barrière et al., 2008; Rossignol et al., 2009). At weeks 1–2 after the first hemisection, only one cat required balance assistance (Table 1) while at weeks 7–8, no cat required balance assistance. After the second hemisection, all cats required balance assistance at both time points. Although hindquarter weight support was present in all cats after both hemisections, maintaining posture was challenging after the second. Weight support can be controlled at a spinal level whereas postural control requires supraspinal inputs (Macpherson et al., 1997). Thus, remaining pathways transmitting signals from supraspinal structures and potentially new ones bridging the lesions, such as short propriospinal pathways, are insufficient to restore postural control.

Although all cats recovered quadrupedal locomotion after staggered hemisections, some cats required perineal stimulation after the second hemisection (Table 1), which increases spinal neuronal excitability and facilitates hindlimb locomotion in spinal mammals through an undefined mechanism (Eidelberg et al., 1980; Alluin et al., 2015; Harnie et al., 2019; Merlet et al., 2021; Audet et al., 2022). Previous studies proposed that the amount of locomotor training constitutes an important factor in locomotor recovery after partial spinal lesions (Kloos et al., 2005; Rossignol et al., 2009). We recently showed that hindlimb locomotor recovery in spinal cats occurs largely spontaneously without task-specific training (Harnie et al., 2019). In the present study, although cats did not receive treadmill training after staggered hemisections, they performed various tasks that can be considered training (see Materials and Methods). Cats were also free to move in their cage and in a dedicated room. They could have developed compensatory behavioral strategies through self-training and some cats are naturally more active and athletic than others. We think that having cats perform a variety of locomotor tasks provided a baseline level of physical activity that reduced interanimal variability, although some cats remained considerably more active than others before and after SCI. The greatest source of variability is undoubtedly because of the personality, motivation, and natural athleticism of individual cats.

### Interlimb coordination is different, weaker, and more variable

We observed 2:1 fore-hind coordination after the first and second hemisections where one forelimb performs two cycles within a hindlimb cycle, as shown previously (Eidelberg et al., 1980; Kato et al., 1984; Howland et al., 1995; Jiang and Drew, 1996; Brustein and Rossignol, 1998; Barrière et al., 2010; Alluin et al., 2011; Górska et al., 2013; Thibaudier et al., 2017). Intact cats also perform 2:1 fore-hind coordination on a transverse split-belt treadmill when the forelimbs step faster than the hindlimbs (Thibaudier et al., 2013; Thibaudier and Frigon, 2014). This led to the hypothesis that forelimb CPGs have an intrinsically faster rhythmicity than hindlimb CPGs (Thibaudier et al., 2017), which is supported by findings in neonatal rats (Juvin et al., 2005). The 2:1 fore-hind coordination after incomplete SCI could result from reduced inhibition from hindlimb to forelimb CPGs (Górska et al., 2013; Frigon, 2017; Thibaudier et al., 2017); whereby reduced inhibition following thoracic SCI releases the intrinsically faster rhythmicity of forelimb CPGs. Disrupting serotonergic spinal pathways in intact rats also produces 2:1 fore-hind coordination (Sławińska et al., 2021). Functionally, 2:1 coordination could represent a strategy to maximize static and dynamic stability (Thibaudier et al., 2017). Performing smaller steps keeps the center of gravity within the support polygon (Cartmill et al., 2002). Another functional reason could be to avoid interference of forelimbs and hindlimbs (Fig. 7). The greater distance between the forelimbs and hindlimbs also increases the support polygon. To avoid interference, cats often adopt pacing on a treadmill where homolateral limbs move in phase (Blaszczyk and Loeb, 1993). However, after incomplete SCI, cats might not be able to transition to a pacing gait.

We showed weaker and more variable fore-hind coordination after staggered hemisections by measuring the phasing between right forelimb and right hindlimb contacts and by performing circular statistics (Fig. 3; Table 3). Weaker fore-hind coordination is consistent with previous studies in rats and cats (Kato et al., 1984; Stelzner and Cullen, 1991; K.C. Murray et al., 2010; Cowley et al., 2015). The second hemisection did not produce significant additional effects in terms of step-by-step consistency of fore-hind coordination (Table 3). However, it is important to note that cats required balance assistance after the second hemisection and providing this aid undoubtedly facilitated fore-hind coordination. Impaired coordination between the forelimbs and hindlimbs could be because of lesioned propriospinal pathways between cervical and lumbar levels and the disruption of direct supraspinal pathways to the lumbar cord (Sherrington and Laslett, 1903; English, 1980; Kato et al., 1984; Bareyre et al., 2004; Courtine et al., 2008). The loss of interlimb reflex pathways also could have contributed to impaired fore-hind coordination (Hurteau et al., 2018). Several pathways from the brain can evoke responses in the four limbs during locomotion, as shown in cats for reticulospinal (Drew and Rossignol, 1984; Drew, 1991), corticospinal (Rho et al., 1999; Bretzner and Drew, 2005), rubrospinal (Rho et al., 1999) and vestibulospinal (Matsuyama and Drew, 2000a, b) pathways (for review, see Frigon, 2017).

Support periods reorganized after staggered hemisection (Fig. 5). Periods of triple support involving the two hindlimbs and one forelimb decreased after the first hemisection and remained decreased after the second. Periods of triple support involving the right hindlimb and both forelimbs increased after the second hemisection. Left homolateral support also decreased after the second hemisection, suggesting a shift in weight support away from the lateral hemisection on the left side. When both forelimbs contact the ground, they provide greater stability. Both diagonal support periods increased after the second hemisection. Although the cat is most unstable in diagonal support, these phases help propel the body forward, increasing quadrupedal locomotion efficiency (Farrell et al., 2014). Thus, increased diagonal support and triple support involving the forelimbs could be a strategy to facilitate forward movement while maintaining stability after staggered hemisections.

### Spinal sensorimotor circuits play a prominent role in hindlimb locomotor recovery

Many mammals recover hindlimb locomotion after complete spinal transection because the spinal locomotor CPG can still interact with sensory feedback from the hindlimbs (Shurrager and Dykman, 1951; Lovely et al., 1986, 1990; Barbeau and Rossignol, 1987; Bélanger et al., 1996; De Leon et al., 1998, 1999; Leblond et al., 2003; Cha et al., 2007; Harnie et al., 2019). Barrière et al. (2008) demonstrated that the spinal locomotor CPG makes an important contribution to hindlimb locomotor recovery following incomplete SCI. They showed that cats that had recovered following an incomplete SCI at T10–T11 expressed hindlimb locomotion the day after a spinal transection at T13–L1, which normally takes a minimum of two to three weeks if only a spinal transection is performed. These results have been confirmed in other studies (Frigon et al., 2009; Barrière et al., 2010; Martinez et al., 2011, 2012). Here, we extend these results by showing that hindlimb locomotion was expressed the day following a spinal transection made 9–10 weeks after the second hemisection (Fig. 8). This indicates that the spinal network controlling the hindlimbs had already undergone plastic changes after staggered hemisections, making it more independent from descending signals originating above the lesions. Changes in the spinal cord can include intrinsic changes in neuronal excitability (K.C. Murray et al., 2010) and/or in sensorimotor interactions from peripheral afferents (Frigon et al., 2009; Gossard et al., 2015). Kato et al. (1984) observed that hindlimb movements were initiated following forward movement induced by the forelimbs after staggered hemisections, much like a pantomime horse. Signals from muscle and/or cutaneous afferents likely play a major role in initiating hindlimb movements after staggered hemisections. This is not to say that descending signals cannot still influence and control the lumbar CPG through new short relay propriospinal pathways (Cowley et al., 2015).

### Locomotor recovery involves a series of neuroplastic changes

**Figure 9.**
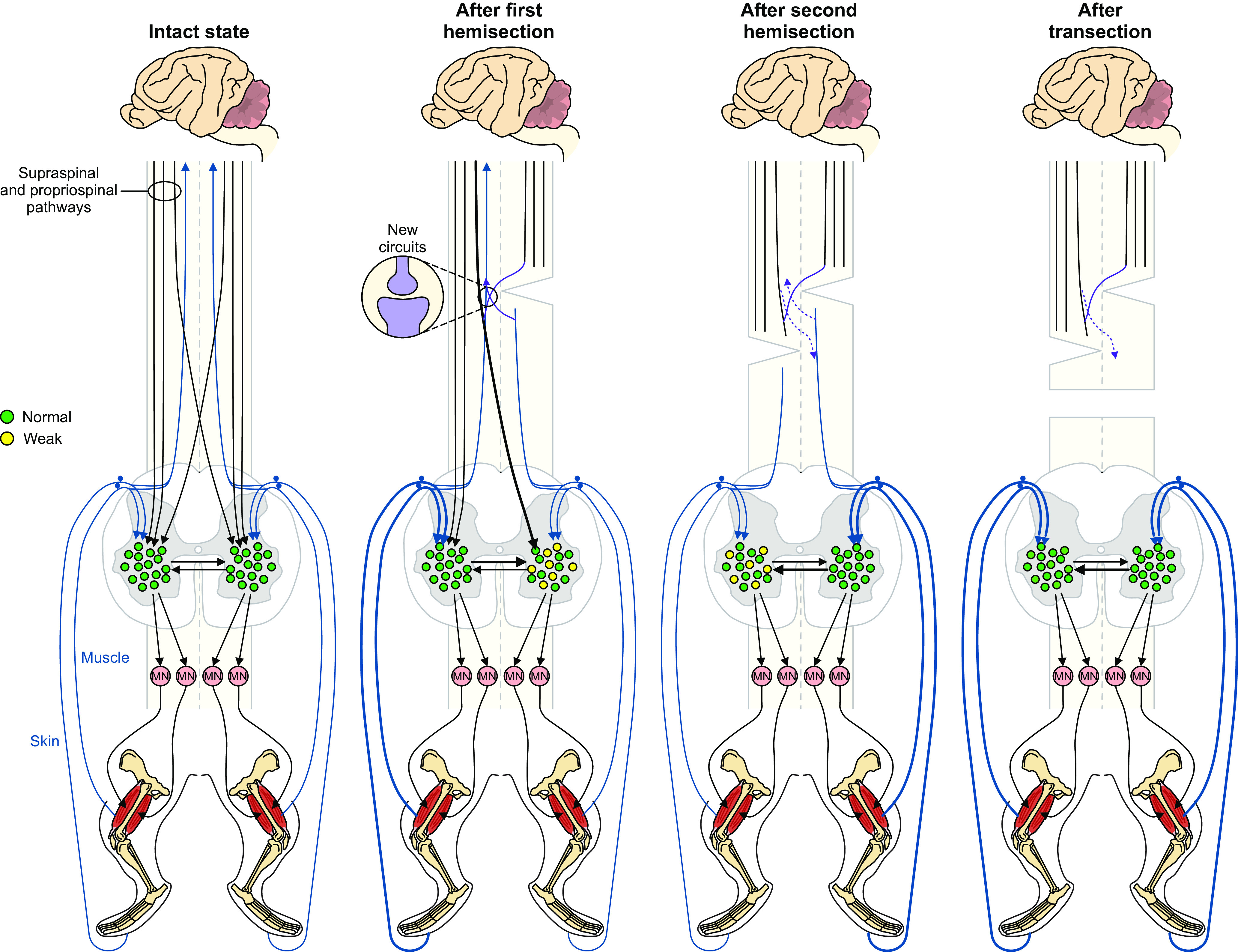
Potential changes in spinal sensorimotor circuits after staggered hemisections. In the intact state, descending supraspinal and propriospinal pathways reach lumbar spinal interneurons that control spinal motoneurons. Pathways transmitting signals from proprioceptive and cutaneous afferents ascend to the brain and project locally to spinal interneurons. After the first hemisection performed on the right side, ipsilesional lumbar neurons have weaker activity and increased weight support of the contralesional left hindlimb increases load feedback from extensors and cutaneous afferents. Thicker lines represent increased influence. The left spinal network increases its influence on the right spinal network. New descending and ascending pathways also form to facilitate communication between the brain and spinal cord. After the second hemisection performed on the left side, neurons of the right spinal network have recovered their activity following the first hemisection. Direct ascending and descending pathways are disrupted but new pathways can form through short propriospinal relays. After spinal transection, both the left and right spinal networks function without descending inputs and hindlimb locomotion is expressed, possibly via strengthened sensorimotor interactions bilaterally.

As mentioned above, we observed several changes in the locomotor pattern. Figure 9 schematically illustrates potential changes in spinal sensorimotor circuits after staggered hemisections involved in locomotor recovery based on left-right asymmetries in cycle and phase durations (Fig. 4*C*) and the immediate expression of hindlimb locomotion after spinal transection (Fig. 8). After the first hemisection, ipsilesional lumbar neurons have weaker activity and longer stance phases and increased weight support of the left hindlimb increases load feedback from extensors and cutaneous afferents. The left spinal network increases its influence on the right spinal network. Anatomical and functional asymmetric changes take place within the spinal cord (M. Murray and Goldberger, 1974; Hultborn and Malmsten, 1983; Helgren and Goldberger, 1993; Frigon et al., 2009). New descending and ascending pathways also form to facilitate descending commands from and to the brain (Fouad et al., 2000; Raineteau et al., 2002; Ballermann and Fouad, 2006; Courtine et al., 2008; Ghosh et al., 2010; Rosenzweig et al., 2010). However, these are insufficient to restore fore-hind coordination. After the second hemisection, neurons of the right spinal network have recovered their activity and stance and weight support is longer/increased for the right hindlimb. The right spinal network increases its influence on the left one. Direct ascending and descending pathways are disrupted but new pathways can form through short propriospinal relays (Zaporozhets et al., 2006; Cowley et al., 2008). However, these are insufficient to restore postural control. Over time after the second hemisection, spinal neuronal activity controlling the left hindlimb recovers. After spinal transection, both left and right spinal networks function without descending inputs and hindlimb locomotion is expressed, possibly via strengthened sensorimotor interactions bilaterally.

In conclusion, staggered hemisections constitute an interesting SCI paradigm to investigate the recovery of posture, interlimb coordination, and locomotion. We are currently investigating interlimb reflexes after staggered hemisections and their contribution to postural and locomotor recovery. Future studies need to determine what ascending and descending signals can be transmitted through such lesions, and importantly, if they make meaningful contributions to locomotion and how we can facilitate them using therapeutic approaches.

## Synthesis

Reviewing Editor: David Schoppik, New York University Decisions are customarily a result of the Reviewing Editor and the peer reviewers coming together and discussing their recommendations until a consensus is reached. When revisions are invited, a fact-based synthesis statement explaining their decision and outlining what is needed to prepare a revision will be listed below. The following reviewer(s) agreed to reveal their identity: NONE. Note: If this manuscript was transferred from JNeurosci and a decision was made to accept the manuscript without peer review, a brief statement to this effect will instead be what is listed below.

After reviewing the rebuttal letter and manuscript I think that all of the reviewers’ concerns have been addressed as merited and the manuscript is clearly stronger. I have one concern / suggestion. In Figures 4-7, the p values reported are those resulting from the KW test. However, the lines/asterisks indicate post-hoc tests that reached the corrected significance criteria. I recommend putting the p values into a table to minimize this confusion.

## Author Response

Reviewer #1 (Rationale for Significance Rating for Authors):

None of the results were surprising, making this reviewer wonder what new insights were gained from this study. Nonetheless, there is a value in characterizing kinematic data in detail, even if the results are all expected and confirm the field’s understanding.

Reviewer #1:

Audet et al. use time-lapsed staggered lateral hemisections and a complete transection to characterize interlimb coordination between each lesion. The series of analyses described kinematic adaptations occurring after each lesion and what can and cannot be compensated or recovered after disrupting descending supra- and propriospinal pathways. None of the results were surprising, making this reviewer wonder what new insights were gained from this study. Nonetheless, there is a value in characterizing kinematic data in detail, even if the results are all expected and confirm the field’s understanding. Whether such nature of a manuscript meets the expectation of the Journal of

Neuroscience is up to the editors’ decision.

While we accept the Editors’ decision, it is not clear why results need to be surprising and unexpected to provide insight and be worthy of publication. Most results, even those published in Nature and Science, can be predicted based on the literature.

That said, I find the manuscript an extremely laborious read. I listed the following suggestions to improve the readability. - Journal of Neuroscience manuscripts should be written for the diverse background of neuroscientists. However, the authors use phrases in the abstract and throughout the main text 1:2 (or 2:1 patterns), support periods, etc., terms that perhaps only the Frigon lab members and close collaborators understand immediately. Authors will benefit from avoiding using those terms in the abstract and defining them early on without waiting until well into the manuscript.

The 2:1 fore-hind coordination was defined upon first use in the introduction, but we now repeat it in the introduction. The 2:1 characterization is included in motor impairment scales to quantify locomotor recovery, such as the BBB scale. Everyone studying locomotor recovery in quadrupedal preclinical models is aware of such coordination patterns. We inserted additional definitions in the Results section and in Figure 2 legend. It should be clearer now.

We do not understand what is unclear about support periods, which was defined in the Methods and have been used in both human (single and double support) and animal research for decades. We even have diagrams showing the support periods. We added an extra definition for support periods (i.e. when two, three or four limbs are in contact with the treadmill surface).

- The text often refers to the entire figure, and accompanying figure legends are so minimal that it was challenging to navigate through the paper. Please break down the text to each figure panel. The same goes for figures. Figures 2, 3, 6, and 8 are data figures that do not contain subpanels. These are difficult to navigate without authors guiding readers through the main texts to individual figure panels.

We broke down the panels of Figures 2, 3 (new figure) and 7 (old figure 8). Legends have been expanded.

For Figure 5 (old Figure 6) all periods of support were/are numbered above each panel.

- I find Figure 3 incomprehensible as is. What does the left column represent? The authors should have explained the significance of the first and second steps and why they separate these two and none others.

2

Figure 3 was removed and replaced by polar plots. We do not know which column the Reviewer was referring to. The old Figure 3 did not have columns. The significance of the first and second forelimb cycles is now explained in more detailed. There are no other cycles to separate.

- I find reference to exceptions to the data (one out of 8 animals did not recover.... Etc) distracting. This is not to say that transparency isn’t essential. I suggest the authors focus on the main findings and messages and include details at the end of each section or perhaps in figure legends.

This is a good suggestion. We streamlined the section and focused on the main findings (what most cats did after the first and second hemisections).

- The authors reference the same paper, Frigon 2017, twice, once in the introduction and once in the discussion, to insinuate two opposing understandings of the field, i.e., the supraspinal role in FL-HL coordination is unknown vs. argues that they are required. Using the same paper and insinuating two different views comes off as too self-serving. Be consistent.

We do not think that terms like ‘insinuate’ or ‘self-serving’ belong in a peer-reviewed evaluation. This was not our intent. The statement was removed in the Introduction and in the Discussion. It is not clear how these are two opposing understandings. Supraspinal contributions appear to be required because coordination of the fore- and hindlimbs is weakened/impaired after incomplete SCI or was not measured after cervical spinal transection in decerebrate cats. We do not know the specific contributions of various supraspinal pathways to forelimb-hindlimb coordination, other than electrically stimulating these pathways can elicit responses in the four limbs. Some additional text was added to the Introduction and

Discussion to clarify our arguments.

Reviewer #2 (Rationale for Significance Rating for Authors): Audet et al. study the impact of delayed staggered hemisections on the locomotor recovery of cats with an emphasis on fore-hindlimb coordination. Using kinematic analyses in freely walking cats, they describe changes in locomotor pattern and rhythm following delayed staggered lateral thoracic hemisections at different thoracic segments. Ultimately, they show recovery of hindlimb locomotion within 24 hours after a lower thoracic transection.

Animals can recover locomotor functions within 24 hours after a complete low thoracic section following an initial lateral hemisection (Barriere et al., 2008, 2010; Martinez et al., 2011, 2013), thus identifying the role of sensorimotor circuits in hindlimb locomotor recovery. Therefore, the strength of the current paper is the thorough description of time course changes in locomotor parameters following delayed staggered hemisections. Although this will be an interesting paper for the locomotor field, the very descriptive format and the lack of neural mechanisms would fit better in a more specialized journal. Data analysis is limited to kinematic; electromyographic recordings have been performed, but will only be presented in upcoming papers. There is also concern about the experimental design. Some animals have been exposed to a wide range of manipulations (including peripheral nerve cuff implantation, electrical stimulation to induce stumbling corrective responses, and/or environmental manipulations) that could have impacted recovery and generate variability in the data.

We thank the Reviewer for the constructive feedback. You will find answers to the comments below.

Reviewer #2:

Introduction. Several articles should be mentioned regarding complete SCI; e.g., Leblond et al., 2003 for SCI mice and other references for SCI rats.

3

We agree, some additional references were missing. We added three in mice or rats (Leblond et al. 2003; Alluin et al. 2011; Slawinska et al. 2012). Line 86: about the FL-HL coordination in absence of supraspinal inputs, the authors could mention in vitro studies using isolated spinal cord preparations from rodents.

Several sentences were added:

The isolated spinal cord of neonatal rats also produces quadrupedal-like rhythmic activity, as demonstrated by recording the activity from cervical and lumbar roots during drug-induced fictive locomotion (Juvin et al., 2005, 2012). In these neonatal rat preparations, the activity recorded from cervical and lumbar roots is coordinated, consistent with propriospinal pathways coupling cervical and lumbar CPGs (Juvin et al., 2005). Similar demonstrations have not been made in adult preparations and as stated, in high spinal decerebrate cats during treadmill locomotion. In real locomotion on a treadmill or overground, the loss of supraspinal inputs and postural control might prevent coordinated fore- and hindlimb movements.

Line 99 and 108: format Kato et al. (1984)

Yes, corrected.

Line 180: experimental protocol. In order to reduce the number of animals used, all cats used in the current study participated in other studies that included implantation of nerve cuffs for electrical stimulation of different peripheral nerves. Some cats were stimulated before and after the 1st hemisection with long trains of stimulation to induce stumbling forelimb and hindlimb corrections during locomotion. There were different types of training: split-belt treadmill locomotion, stepping over obstacles, and a different surface (foam). All these surgical, training, stimulation, and environmental manipulations could have an impact on the normal recovery of animals and induce variability.

Yes, this undoubtedly played a role, but we think this reduces inter-animal variability. After the lesions, some animals are naturally more active than others based on their personality, motivation, athleticism, etc. Having them do a variety of tasks provides a baseline level of physical activity for all cats even though some remain considerably more active than others. We added the following sentence to the

Discussion:

We think that having cats perform a variety of locomotor tasks (see Experimental protocol in Materials and Methods) provided a baseline level of physical activity that reduced inter-animal variability, although some cats remain considerably more active than others before and after SCI. The greatest source of variability is undoubtedly due to the personality, motivation, and natural athleticism of individual cats.

Line 230: EMG recordings will be only used for illustrative purpose and not analyzed. Why will EMG analyses be presented only in upcoming papers, whereas they could be integral to the current study? We agree that providing EMG results (other than for illustrative purposes) would have been interesting but there are reasons why we did/could not. A limitation of the chronic recordings over several months is that we cannot be certain that properties of the recording electrodes or at the recording sites did not change over time. One way to circumvent this problem is to normalize EMG data at the different time points. We are currently doing this with other papers in preparation where we compare EMG at different speeds (we can normalize data to a speed and compare the speed modulation across time points) or at different left-right speeds on a split-belt treadmill (we can normalize data to a condition, such as tied- or split-belt). In the present study, we cannot normalize our data at a given time point because we only have one speed in one condition. Another problem is that some cats only display 2:1 coordination patterns after the first or second hemisection. Thus, we cannot compare EMG data (burst durations or 4 amplitudes) with the intact state, where only 1:1 coordination patterns were found. EMG data/results will be central in our upcoming papers.

Line 253: I don’t understand why the authors used a coefficient of variation to analyze phase dispersion in Figure 4 and a Rayleigh in Table 3. The presentation is to some extent redundant as both illustrate phase variability. Between the two, circular statistics should be preferred to analyze the variability of a periodic movement such as locomotion.

We deleted Figure 4 and replaced Figure 3 with a circular plot, which goes with Table 3.

Results section. Although the results section describes in depth the changes occurring following the 1st and 2nd lateral hemisection, the section is very systematic and a bit too descriptive. It could be shortened.

We streamlined the Results. Some figures were removed or replaced. Table 4 and accompanying text was removed.

Why did the authors analyze the data at weeks 1-2 for acute recovery after the 1st hemisection, and at weeks 1-4 after the 2nd hemisection? Why not use the same period? Figure 1 illustrates only the lesion extent; it could be a supplementary figure.

It was mentioned in the text. All cats recovered quadrupedal locomotion at weeks 1 or 2 after the first hemisection but after the second hemisection, the first time point where we could obtain stable quadrupedal locomotion was at 3 or 4 weeks.

We prefer to keep lesion extent as a Figure. It is an important result. Figure 3: The frequency of occurrence in Fig, 3 does not add much information. The proportion of occurrence of 2:1 versus 1:1 could be reported. Figures 2 and 3 could be combined.

We deleted Figure 3 and replaced it with circular plots. The proportion of 2:1 versus 1:1 was already shown in Table 2.

Figure 4: The phase interval should be shown for all 6 interlimb couplings. Why present both the coefficient of variation of the phase interval (Fig. 4B) and the Rayleigh (Table 3)? Given that locomotion is a periodic movement, the Rayleigh in Table 3 will be more accurate than the coefficient of variation.

We deleted figure 4. Showing phase intervals for all six interlimb couplings would add too much data that might not be all that pertinent because some cats only performed 2:1 coordination after the first or second hemisection. We would have to separate all 1:1 and 2:1 patterns and have five additional tables for the other couplings. We focused on right homolateral coupling to characterize the coordination between the fore- and hindlimbs. The other couplings (left homolateral and both diagonals) are equivalent because the left and right sides maintain 1:1 coordination at shoulder (forelimb coupling) and pelvic (hindlimb coupling) girdles.

Figure 5: Overall, the forelimb cycle, stance, and swing duration were significantly decreased only after the 2nd hemisection, whereas changes were far more modest and more inconsistent for the left and right hindlimbs. Could this variability reflect the variety of experiences the animals were exposed to (i.e., peripheral nerve cuffs, electrical stimulation, stumbling corrective responses, and/or different ground surfaces)?

See answer to previous comment with regards to experiences/variability.

5

Line 456: Given the negative asymmetric index after the 1st hemisection for the hindlimb stance, I assume that the left-right asymmetries were calculated by subtracting left limb durations from right limb durations and not the way around. However, the asymmetric index matches for the hindlimb swing! Despite no obvious changes in hindlimb cycle, swing, and stance duration, the asymmetry index between left versus right hindlimbs showed significant changes during the stance and swing phase (Fig. 5C), thus arguing for a switch in an asymmetrical locomotor pattern after staggered hemisections favoring a transfer of weight from the left side after the 1st hemisection to the right side following the 2nd hemisection. This could be mentioned in the text. No, we were correct. It is right limb minus left limb durations. After the first hemisection on the right side, right stance duration is shorter than left stance duration, which gives a negative value. The opposite is found for swing duration (right swing > left swing). Yes, the Reviewer is correct that there is a transfer of weight to the left side after the first hemisection to the right side after the second.

We added the following sentence:

This suggests that weight support of the hindquarters shifted to the left contralesional side after the first hemisection and to the right side after the second hemisection of the left spinal cord.

Line 437-445: The authors should report the frequency of occurrence of 2:1 versus 1:1 FL-HL coupling to support their statement at line 446.

Yes, we added: (see Table 2).

Line 488: I have some problems with the interpretation of data in Fig. 6. In comparison to the control, periods 4 and 8 do not support the conclusion that staggered hemisections initially favor support away from the ipsilesional hindlimb following the 1st hemisection. However, periods 3 and 8 suggest a transfer of support from the left side after the 1st hemisection to the right side after the 2nd hemisection (similarly to Fig. 5C).

The Reviewer is correct. We changed to:

Therefore, cats adjust their support periods to maintain dynamic balance during quadrupedal locomotion after staggered hemisections, mainly by decreasing periods of triple support involving both hindlimbs and one forelimb early after the first hemisection (on the right side), and then away from the left hindlimb after the second hemisection (on the left side).

In contrast to temporal measurements (Fig. 5 and 6), spatial measurements (Fig. 7) showed only obvious changes in hindlimbs after the 2nd hemisection. Other than right hindlimb swing, obvious changes in temporal measurements were also observed after the second hemisection. That is why the asymmetry index is more interesting and revealing in terms of changes in the hindlimb pattern.

Figure 8: The distance between RHL-RFL already increased after the 1st hemisection, whereas the distance between LHL-LFL only increased after the 2nd hemisection, thus suggesting that the initial hemisection impacts the ipsilesional FL-HL distance at contact and liftoff. This again could support the statement about the weight transfer after lesion.

Yes, we added the following sentence:

6

The increase in distance between the right limbs after the second hemisection could also improve balance by increasing the support polygon.

Figure 9: I don’t see the 1:2 fore-hind coordination in 9B. What about changes in spatial and temporal locomotor parameters after full transection?

This was our mistake. The stance phases for the limbs were in the wrong order after transection. This has been corrected. We did not show spatial and temporal parameters after full transection because we did not have enough cats for a statistical analysis. The main message is that cats recover hindlimb locomotion the day after the transection.

Discussion

Lines 579 and 628: “the first hemisection weakened fore-hind coordination and made it more variable, with little additional effect of the second hemisection.” It looks like that there are more changes than reported. Although the homolateral couplings on the left and right sides showed a higher variability after the 1st hemisection, the diagonal couplings (Fig. 4) also showed an increased variability after the 2nd hemisection. Not to mention the changes in FL-HL support (Fig. 6) and FL-HL distance (Fig. 8).

Yes, we made some precisions. Figure 4 was deleted however.

Some key articles using thoracic hemisection followed by a complete lower thoracic transection in cats should be mentioned and better discussed with respect to the current study (Barriere et al., 2008, 2010; Martinez et al., 2011, 2013).

The Barrière et al. (2008) was already mentioned. We added the following sentences and references: Barrière et al. (2008) demonstrated that the spinal locomotor CPG makes an important contribution to hindlimb locomotor recovery following incomplete SCI. They showed that cats that had recovered following an incomplete SCI at T10-T11 expressed hindlimb locomotion the day after a spinal transection at T13-L1, which normally takes a minimum of 2-3 weeks if only a spinal transection is performed. These results have been confirmed in other studies (Frigon et al., 2009; Barrière et al., 2010; Martinez et al., 2011, 2012).

Line 634: “(Frigon, 2017) argued that fore-hind coordination requires supraspinal commands.” It will be important to develop this a bit more...

The statement was deleted and additional details are provided.

Line 645: Barrière et al., (2008) and others have previously shown that animals that have recovered locomotion from an hemisection can recover bilateral hindlimb locomotion within 24 hours after a complete transection.

Yes, this was mentioned. We added some text and references. See answer to previous comment.
